# Acyl-CoA-dependent and acyl-CoA-independent avocado acyltransferases positively influence oleic acid content in nonseed triacylglycerols

**DOI:** 10.3389/fpls.2022.1056582

**Published:** 2023-01-11

**Authors:** Jyoti Behera, Md Mahbubur Rahman, Jay Shockey, Aruna Kilaru

**Affiliations:** ^1^ Department of Biological Sciences, East Tennessee State University, Johnson City, TN, United States; ^2^ dNTP Laboratory, Teaneck, NJ, United States; ^3^ U.S. Department of Agriculture, Agricultural Research Service, Southern Regional Research Center, Commodity Utilization Research Unit, New Orleans, LA, United States

**Keywords:** triacylglycerol, avocado, nonseed, oleic acid, DGAT1, DGAT2, PDAT1

## Abstract

In higher plants, acyl-CoA:diacylglycerol acyltransferase (DGAT) and phospholipid:diacylglycerol acyltransferase (PDAT) catalyze the terminal step of triacylglycerol (TAG) synthesis in acyl-CoA-dependent and -independent pathways, respectively. Avocado (*Persea americana*) mesocarp, a nonseed tissue, accumulates significant amounts of TAG (~70% by dry weight) that is rich in heart-healthy oleic acid (18:1). The oil accumulation stages of avocado mesocarp development coincide with high expression levels for type-1 DGAT (*DGAT1*) and *PDAT1*, although type-2 DGAT (*DGAT2*) expression remains low. The strong preference for oleic acid demonstrated by the avocado mesocarp TAG biosynthetic machinery represents lucrative biotechnological opportunities, yet functional characterization of these three acyltransferases has not been explored to date. We expressed avocado *PaDGAT1, PaDGAT2*, and *PaPDAT1* in bakers’ yeast and leaves of *Nicotiana benthamiana*. *PaDGAT1* complemented the TAG biosynthesis deficiency in the quadruple mutant yeast strain H1246, and substantially elevated total cellular lipid content. *In vitro* enzyme assays showed that *Pa*DGAT1 prefers oleic acid compared to palmitic acid (16:0). Both *PaDGAT1* and *PaPDAT1* increased the lipid content and elevated oleic acid levels when expressed independently or together, transiently in *N. benthamiana* leaves. These results indicate that *Pa*DGAT1 and *Pa*PDAT1 prefer oleate-containing substrates, and their coordinated expression likely contributes to sustained TAG synthesis that is enriched in oleic acid. This study establishes a knowledge base for future metabolic engineering studies focused on exploitation of the biochemical properties of *Pa*DGAT1 and *Pa*PDAT1.

## 1 Introduction

In plants, storage lipids primarily consist of triacylglycerol (TAG) that are assembled through three sequential acylations of glycerol-3-phosphate (G3P), catalyzed by *sn*-specific membrane-bound acyltransferases (Shockey et al., 2006; McFie et al., 2010; Turchetto-Zolet et al., 2011) in the endoplasmic reticulum (ER) membrane. The first two acylations are catalyzed by glycerol-3-phosphate acyltransferase and lysophosphatidic acid acyltransferase to form an important metabolic intermediate phosphatidic acid (PA) (Murata and Tasaka, 1997; Shockey et al., 2016). After removal of a phosphate group from the *sn*-3 position of PA by phosphatidic acid phosphatase, diacylglycerol (DAG) is acylated by either diacylglycerol acyltransferase (DGAT) or phospholipid:diacylglycerol acyltransferase (PDAT) (Banaś et al., 2000; Dahlqvist et al., 2000) to yield TAG. At least two structurally unrelated polypeptides, namely DGAT1 and DGAT2, can catalyze the transfer of a fatty acid from acyl-coenzyme A (CoA) to DAG to generate TAG (Hobbs et al., 1999; Routaboul et al., 1999; Shockey et al., 2006; Xu et al., 2014). The specific roles of the two major DGAT types are not fully established, although existing studies suggest that DGAT1 plays a major role in seed and fruit oil accumulation in many plant species, whereas DGAT2 often plays a larger role in TAG production in certain novel oilseeds that produce high levels of unusual fatty acids (Burgal et al., 2008; Li et al., 2010a; Li et al., 2010b; Li et al., 2012; Chen et al., 2022). More recently, a third category of *DGAT* genes that encode soluble cytoplasmic activity, called DGAT3, were also shown to be associated with oil metabolism in plants (Saha et al., 2006; Hernández et al., 2012; Cao et al., 2013; Aymé et al., 2018; Xue et al., 2022). PDAT, on the other hand, catalyzes the transfer of an acyl group from phosphatidylcholine (PC) to the *sn*-3 position of DAG to form TAG (Dahlqvist et al., 2000). *In vitro* PDAT activity assays suggest a modest preference for the *sn*-2 position of acyl donor PC, although transfer from the *sn*-1 position also occurs (Ståhl et al., 2004). PC is of particular importance in oilseed metabolism; the ER membrane PC pool serves as the substrate for desaturation by fatty acid desaturase FAD2 and FAD3 in vegetative and reproductive organs of plants (Ohlrogge and Browse, 1995; Bates et al., 2007; Bates et al., 2009), as well as modifications such as fatty acid hydroxylation, conjugation, and epoxidation found in various exotic oilseeds (Bates et al., 2009). As such, direct metabolic utilization of PC by PDAT is a major determinant in the distinctive seed fatty acid profile of hydroxy-, and epoxy-fatty acid accumulating plant species such as *Ricinus communis* (castor bean), *Vernonia galamensis*, *Euphorbia lagascae*, and *Stokesia laevis* (Li et al., 2010a; van Erp et al., 2011). Along with DGAT, PDAT activity also plays an important complementary role in oil content and fatty acid composition in plants with otherwise ‘normal’ seed fatty acid profiles, such as *Arabidopsis thaliana* and *Camelina sativa* (Ståhl et al., 2004; Zhang et al., 2009; Marmon et al., 2017).

DGAT1 was first identified in mouse, based on sequence similarity with the mouse acyl CoA:cholesterol acyltransferase, and was shown to possess acyl-CoA-dependent DAG acyltransferase activity (Cases et al., 1998). Subsequently, DGAT1 homologs were identified in many plant species (Hobbs et al., 1999; Routaboul et al., 1999; Zou et al., 1999; Bouvier-Navé et al., 2000; He et al., 2004; Shockey et al., 2006; Aymé et al., 2015). DGAT1 proteins are integral ER membrane proteins containing between six and ten transmembrane-spanning domain (TMDs) (Yen et al., 2008). Both termini of the tung DGAT1 protein are cytosol-exposed, indicating that all or most DGAT1s (at least in plants) contain an even number of TMDs (Shockey et al., 2006; Zhang et al., 2009). The variable *N*-terminal region of DGAT1 is cytosol-localized and is associated with distinct functions in different organisms (Liu et al., 2012). Overall, DGAT1 orthologs (~490-530 amino acid residues) contain several conserved motifs including a DAG binding motif, an acyl-CoA binding motif, a hydrophobic *C*-terminal ER retrieval motif, and a fatty acid-binding protein signature (McCartney et al., 2004; Shockey et al., 2006; Guihéneuf et al., 2011). A relatively smaller (~320-350 amino acid residues) protein with two TMDs (Shockey et al., 2006), DGAT2 arose by convergent evolution, and is structurally unrelated to the DGAT1 family. First purified from the oleaginous fungus *Mortierella ramanniana* (Lardizabal et al., 2001), DGAT2 also has distinct effects on lipid content and composition in a broad array of organisms, from humans and mice (Cases et al., 2001; Stone et al., 2004) to yeast and castor (Oelkers et al., 2002; Burgal et al., 2008).

First identified in yeast (Dahlqvist et al., 2000), PDAT is a large (~650-680 amino acid residues) protein that is targeted to the ER membrane by a single TMD, rendering much of the protein facing the ER lumen. Overexpression of membrane anchor-deleted forms of yeast PDAT resulted in a soluble secreted protein that retains most of the biochemical properties of the native enzyme (Ghosal et al., 2007). The identity of the PDAT active site(s) remain unknown, but plant orthologs do show homology to the catalytic triad with nucleophile-acid-base (S-D-H) residues typically found in the alpha-beta hydrolase fold motif (Ollis et al., 1992).

Functional characterization of DGAT genes in mammalian systems has mostly outpaced similar research in plants. In mammals, both DGAT1 and DGAT2 contribute to TAG biosynthesis (Cases et al., 1998) and exhibit non-redundant physiological roles (Smith et al., 2000; Chen et al., 2002; Stone et al., 2004), while the specific roles and relative quantitative contributions of DGAT and PDAT to seed oil metabolism is less well-understood. At least six *PDAT*-like genes have been identified in *Arabidopsis* but only *PDAT1* accounted for most of the measurable acyltransferase activity (Ståhl et al., 2004). Disruption or overexpression of *PDAT1* had no significant impact on TAG accumulation in *Arabidopsis* seeds (Ståhl et al., 2004; Mhaske et al, 2005). Loss of DGAT1 activity in *Arabidopsis* causes modest reductions in total seed oil content and a strong shift towards polyunsaturated fatty acids in seed oil (Routaboul et al., 1999; Zou et al., 1999). However, the phenotypic effect of a combined loss of DGAT1 and PDAT1 in *Arabidopsis* is severe; a *pdat1*/*dgat1* double mutant contained abnormal, sterile pollen, resulting in poorly developed nonviable embryos (Zhang et al., 2009). Seed-specific RNAi silencing of *PDAT1* expression in an *Arabidopsis dgat1* mutant background defined the outer limits of tolerable interference to seed oil metabolism, resulting in 70–80% reductions in seed TAG content (Zhang et al., 2009). The ability of *PDAT1* to compensate for the loss or suppression of *DGAT1* and *vice versa* suggests that the acyl-CoA-dependent and -independent pathways are cooperatively involved in TAG synthesis in many oil-rich tissues.

As such, DGAT and PDAT enzymes possess biotechnological potential as tools for metabolic engineering studies. In transgenic studies, *Arabidopsis DGAT1* enhanced TAG content when overexpressed in yeast and tobacco leaves (Bouvier-Navé et al., 2000). Some plant DGAT1 show preference for substrates containing distinct fatty acids (Li et al., 2010a; Li et al., 2010b; Li et al., 2012; Aznar-Moreno et al., 2015; McKeon and He, 2015). Arabidopsis DGAT1 prefers long-chain fatty acids, while oil palm (*Elaeis guineensis*) *EgDGAT1-1* prefers medium-chain fatty acids when expressed in yeast (Aymé et al., 2015). Enrichment of unusual fatty acids in engineered seed oils has been achieved with some plant DGAT2 enzymes. Tung tree (*Vernicia fordii*) DGAT2 preferentially incorporates α-eleostearic acid, a conjugated trienoic C18 fatty acid, in transgenic plants and yeast (Shockey et al., 2006; van Erp et al., 2015), while castor, *V. galamensis*, and *S. laevis* DGAT2s display strong selectivity towards epoxy and hydroxy fatty acids, both *in vitro* and *in vivo* (Kroon et al., 2006; Burgal et al., 2008; Li et al., 2010a; Li et al., 2010b). In addition to the striking differences in biochemical properties, tung and castor *DGAT2* are expressed at much higher levels than their cognate *DGAT1*s in developing seeds, suggesting strong positive selection for roles in selective accumulation of unusual fatty acids (Liu et al., 2012). However, in ‘normal’ seed tissues that accumulate only standard C16-C22 fatty acids, such as the embryos of *Arabidopsis*, rapeseed, and soybean, *DGAT1* was highly expressed, while *DGAT2* transcript was barely detectable (Li et al., 2010b; Troncoso-Ponce et al., 2011; Li et al., 2013; Zhang et al., 2016).

Avocado (*Persea americana*), a basal angiosperm species from the Lauraceae family, is an evolutionarily important cash crop that accumulates a significant amount (60-80% by dry weight) of nutritionally rich TAGs in its mesocarp (nonseed) tissue (Takenaga et al., 2008; Kilaru et al., 2015). In seeds, TAGs typically accumulate in the form of lipid droplets (LDs), which are ordered, spherical, neutral lipid aggregates, surrounded by an oleosin-rich phospholipid/protein monolayer (Huang, 1996). In avocado mesocarp, LDs are associated with a non-oleosin class of proteins and are predominantly composed of monounsaturated oleic acid (18:1) (Gidda et al., 2013; Horn et al., 2013). Although both *PaDGAT1* and *PaDGAT2* are expressed in mesocarp tissue, transcript levels for *PaDGAT1* were dominant, with ≥2-fold higher expression than *PaDGAT2* throughout fruit development (Kilaru et al., 2015). *PaPDAT1* expression was also detected at a comparable level to that of *PaDGAT1* during the early fruit developmental stages (Kilaru et al., 2015). Transcriptome studies of oil-rich nonseed tissues such as avocado, oil palm, and olive revealed several similarities in the expression pattern of genes involved in plastidial fatty acid synthesis, while unique variations were observed in the expression of genes involved in TAG assembly (Bourgis et al., 2011; Troncoso-Ponce et al., 2011; Kilaru et al., 2015; Rahman et al., 2016). The concomitant expression of both *DGAT1* and *PDAT1* in seed tissues is not evolutionarily maintained across all oilseeds; the mesocarp expression patterns might represent a less-common repertoire of coordinated TAG biosynthesis regulation in avocado. However, the acyltransferases from avocado that are involved in TAG assembly have not been identified and functionally characterized to date. Hence, we sought to study the properties of *PaDGAT1, PaDGAT2*, and *PaPDAT1* and assess their potential contributions to TAG biosynthesis in nonseed tissues.

## 2 Materials and methods

### 2.1 Identification and *in silico* analyses of putative *Pa*DGAT1, *Pa*DGAT2, and *Pa*PDAT1

Full-length complementary DNA (cDNA) sequences for *PaDGAT1* (OP727298), *PaDGAT2* (OP727299), and *PaPDAT1* (OP727300) were retrieved from the avocado transcriptome database (Kilaru et al., 2015) and verified against avocado genome sequence data (Ibarra-Laclette et al., 2015; Rendón-Anaya et al., 2019). The cDNA sequences were further analyzed using NetStart1.0 prediction software (http://www.cbs.dtu.dk/services/NetStart/) to check for the locations of probable start codons. The corresponding protein sequences were predicted using the ExPASy protein translation tool (https://web.expasy.org/translate/) and the coding sequence for the longest open reading frame was selected for further use. This resulted in 1608 bp, 996 bp, and 2049 bp long putative full-length protein coding sequences for *PaDGAT1, PaDGAT2*, and *PaPDAT1*, respectively (**Table 1**).

**Table 1:** Bioinformatic analytics for *Pa*DGAT1, *Pa*DGAT2 and *Pa*PDAT1.

*In silico* analyses	*Pa*DGAT1	*Pa*DGAT2	*Pa*PDAT1
**Full length cDNA**	**2079 bp**	**1385 bp**	**2079 bp**
**Coding sequence length**	**1608 bp**	**996 bp**	**2052 bp**
**Probability of start codon**	**78.6%**	**86.0%**	**89.80%**
**Protein length**	**535 aa**	**331 aa**	**682 aa**
**Estimated molecular weight**	**61.2 kDa**	**37.2 kDa**	**75.8 kDa**
**No of transmembrane domains**	**9**	**6**	**1**

The predicted avocado protein sequences were compared to other plant orthologs by multiple sequence alignments using Clustal Omega (https://www.ebi.ac.uk/Tools/msa/clustalo/). Probable transmembrane spanning domains were predicted using TMHMM transmembrane domain prediction software (http://www.cbs.dtu.dk/services/TMHMM/) and Phobius (https://phobius.sbc.su.se/) and were compared with transmembrane domains of characterized DGAT1, DGAT2, and PDAT1 proteins, respectively. The phylogenetic tree was constructed by the maximum likelihood method based on the Poisson correction model (Zuckerkandl and Pauling, 1965), and the tree with the highest log likelihood was shown. The phylogenetic figure was drawn to scale, and evolutionary analyses were performed in Molecular Evolutionary Genetics Analysis (MEGA) X (Kumar et al., 2018). The name of each protein consists of the first letter of the species followed by the first letter of the genus.

The three-dimensional (3D) structures were predicted by SWISS-MODEL interactive workspace (https://swissmodel.expasy.org/interactive) using the 3D structure of proteins showing the highest sequence similarity as templates for each respective protein of interest. The predicted 3D models with full atomic coordination information were obtained as Protein Data Bank (.pdb) files and visualized using USCF CHIMERA 1.14 software (https://www.cgl.ucsf.edu/chimera/cgi-bin/secure/chimera-get.py?file=win64/chimera-1.16-win64.exe). For structure comparison, the ‘MatchMaker > align’ option was used and results were obtained as superimposed structures and structure-based sequence alignments. The positions of structurally important residues are shown in the figures with the amino acid single letter code followed by the corresponding position. The root mean square deviation (RMSD) values of C-alpha atoms were also calculated and shown along with the structure-based sequence alignment.

### 2.2 Cloning of putative *Pa*DGAT1, *Pa*DGAT2, and *Pa*PDAT1 for yeast expression

A bi-directional expression vector (pESC-URA, Agilent, USA) was used to direct galactose-inducible expression of *PaDGAT1*, *PaDGAT2* and *PaPDAT1* in bakers’ yeast (*Saccharomyces cerevisiae*). The cDNAs were synthesized from avocado mesocarp total RNA using oligo (dT) primer and AMV Reverse Transcriptase (Promega). For PCR amplification, forward and reverse primers were designed to include *Not*I and *Pac*I restriction sites, located upstream of the start codon and downstream of the stop codon, respectively. Additionally, “ACC” nucleotides were added upstream of the restriction site in the forward primer to optimize translational efficiency (Kozak, 1989). The full-length cDNAs of *PaDGAT1*, *PaDGAT2*, and *PaPDAT1* were amplified using Advantage 2 PCR (clontech) kit. The resulting PCR products and the vector were digested with *Not*I and *Pac*I (New England Biolabs, USA). Agarose gel-purified digested amplicons and vector DNA were ligated using T4 DNA ligase (New England Biolabs, USA). Aliquots of the ligation reactions were transformed into chemically competent *E. coli* TOP10 cells and selected on solid agar media plates containing the appropriate antibiotic. Positive transformants were selected by colony PCR and further confirmed by sequencing. Plasmid DNA was extracted and used for yeast transformation.

The *S. cerevisiae* quadruple mutant strain H1246 (*Δdga1*/*Δlro1*/*Δare1*/*Δare2*) (Sandager et al., 2002) was transformed either with empty pESC-URA as a negative control or pESC-*VfDGAT*1-URA (*Vernicia fordii* DGAT1) for the positive control (Shockey et al., 2006), or each of pESC-*PaDGAT1*-URA, pESC-*PaDGAT2*-URA, and pESC-*PaPDAT1*-URA, using the S.c. EasyComp Transformation Kit (Invitrogen, USA). These transformed cells were plated on SD-URA (synthetic dextrose minus uracil) plates containing 2% glucose, 0.67% yeast nitrogen base. After 2-4 days, several colonies were selected and induced to grow in SRGG-URA (synthetic raffinose, glycerol, galactose minus uracil) plates containing 1% raffinose, 0.25% glycerol, 2% β-galactose (Difco), 0.67% yeast nitrogen base (Difco), and synthetic complete mixture of amino acids minus uracil for 24 hours. All liquid cultures were grown in similar media at 28°C on a shaker at 250 rpm.

### 2.3 Indirect enzyme activity analysis *via* lipotoxicity rescue assay

Lipotoxicity rescue assays were performed as described previously (Sandager et al., 2002; Garbarino and Sturley, 2009; Siloto et al., 2009). All transformed strains including negative and positive controls were grown on SD-URA plates at 28°C for 2-3 days. Positive colonies were first grown in a small volume (~5 mL) of SD-URA liquid media at 28°C. Cell optical densities (OD) were measured spectrophotometrically at 600 nm (OD_600nm_). Starter cultures were back-diluted in SRGG-URA liquid media at 28°C with shaking overnight to induce transgene expression. Fresh SRGG-URA liquid media was prepared and split in half: one portion containing base components with 0.2% Tergitol detergent alone and one portion containing base components, 0.2% Tergitol, and 1.0 mM free linoleic (18:1) or linolenic (18:2) fatty acids (prepared as 0.5 M stock, in ethanol). Both types of media were warmed at 28°C at least 15-30 minutes prior to adding to the yeast cells to ensure even dispersal of fatty acid in the media, followed by addition of 0.1 OD of pelleted, washed cells into each media type. Cultures were incubated with vigorous shaking for 26 hours at 28°C and cell densities sampled at various time points (0, 12, 22, and 26 hours) until cultures reached late log or stationary phase (~8-12 OD). The cell density readings were plotted against various time points to generate yeast growth curves. Three technical replicates and two biological replicates were done to ensure reproducibility.

### 2.4 Microsome isolation

Microsomes were prepared from transgenic H1246 yeast strains as previously described (Shockey et al., 2006). Briefly, yeast cultures grown overnight in SRGG-URA liquid media were harvested by centrifugation, washed twice with sterile dH_2_0 and once with 1X phosphate buffer saline (PBS) respectively. After washing, the pellets were resuspended in cell lysis buffer I (1x PBS containing Mini EDTA-free protease cocktail tablets from Roche Diagnostics; 1 tablet/50 mL buffer) and transferred to a 15-mL glass tube. An equal volume of 0.50 mm acid-washed glass beads was added to the cells and were lysed by vortexing. Debris and unbroken cells were removed by low-speed centrifugation. The supernatant was recovered and centrifuged again at 100,000 x *g* for 1 h at 4°C. Microsomal pellets from the high-speed spin were collected and resuspended in 50 mM Tris-HCl and 25% glycerol, pH 8.0.

### 2.5 *In vitro* enzyme activity and substrate specificity

Enzyme activity and substrate specificity of DGAT1 were measured *in vitro* by the incubation of microsomal fractions with different combinations of radiolabeled fatty acyl-CoA substrate and DAG as described previously with some modifications (Shockey et al., 2006). Briefly, 25 μg of microsomal membrane proteins were incubated with 10 μM [1- ^14^C] acyl-CoAs [palmitoyl (16:0) -CoA or oleoyl (18:1) -CoA from PerkinElmer, specific activity, 20,000 CPM/μmol] and 400 μM DAG [di-palmitin (di16:0) and diolein (di18:1); stock of 40 mM in methanol)] in a total reaction volume of 100 µL buffered with 100 μM Tris-HCl (pH 8.0). The reaction mixture was incubated at 37°C for one hour in a water bath. Lipids were extracted, concentrated under vacuum, and dissolved in 20 µL chloroform. Lipids were spotted on Silica Gel 60 plates (Whatman) thin layer chromatography (TLC) plates and separated as described previously (Janero and Barrnett, 1981). Developed TLC plates were dried for 10 min and placed on AR-2000 radio-TLC imaging scanner (Bioscan, Inc., USA) to detect and quantify the radioactive TAG spots. Assay mixtures including all components except microsomal proteins were used as a negative control. The *in vitro* assay was performed in triplicate for each acyl-CoA substrate and each enzyme. The obtained data are represented as mean ± SD.

### 2.6 Cloning for *in planta* expression

For *N. benthamiana* (benth) leaf expression, digested *Nco*I/*Sac*II amplicons for *PaDGAT1* and *PaPDAT1* were cloned into the corresponding sites in the pK34 cloning vector. Plasmid K34 contains a *CaMV35S* promoter and a *35S* terminator sequence (Shockey et al., 2015). The recombinant pK34 constructs were selected as described above, followed by *Asc*I digestion and gel-purification of the *CaMV35S promoter:gene:35S terminator* cassettes, and cloning into the corresponding site in the binary expression vector pB110. A viral silencing suppressor protein gene (*P19*) (Wood et al., 2009) was also cloned into pB110, either alone or along with the *DGAT/PDAT* expression cassettes, using the same series of cloning steps and was used for co-infiltration in the benth leaves. Prior to benth leaf infiltration, all positively confirmed plasmids were isolated from *E. coli* and transformed into *Agrobacterium tumefaciens* as described below.

### 2.7 *Agrobacterium*-mediated transformation of *N. benthamiana* leaves

*Agrobacterium* LBA4404 strain was used for transient gene expression in benth leaves. Approximately 100-1000 ng of finished binary plasmid DNA containing the genes of interest were transformed into competent cells, and selected on solid media containing kanamycin as described previously (Behera et al., 2021). Plates were incubated for 3-6 days at 28°C for colony formation. Positive colonies were used to grow O/N starter cultures at 28°C with shaking (250 rpm). Overnight cultures were supplemented with 100 mM acetosyringone and grown for another 2 hours. Pellets were collected by centrifugation at 400 x *g* for 5 min and resuspended in the infiltration buffer (5 mM MgSO_4_, pH 5.7, 100 mM acetosyringone, and 5 mM methyl ethanesulfonate) to a final OD of 0.3.

*N. benthamiana* plants were grown in a 24°C plant growth room with overhead lighting using 9:15 light: dark cycle. Three slightly aged leaves located slightly below the apical bud were selected from five/six-week**-**old *N. benthamiana*. Two leaves were infiltrated at high coverage with each transgenic *A. tumefaciens* strain. The last leaf was used for two controls- mock (containing only infiltration buffer) and negative control, which contains infiltration buffer and *Agrobacterium* cells expressing empty plasmid vector. To facilitate infiltration, several well-spaced small nicks were created with a needle in the abaxial leaf epidermis. After infiltration, plants were then transferred into the growth chamber and allowed to grow for 6-7 days to express the protein.

### 2.8 Yeast cell and benth leaf lipid extraction

Harvested yeast cells were washed twice with sterile distilled water and once with 1X PBS. For infiltrated *N. benthamiana* leaves, samples were collected at 6-7 days after infiltration and 200 mg fresh weight (FW) was used for lipid extraction. Cells/leaves were treated with isopropanol at 80°C for 10-15 min to stop lipolytic activity. Lipid extraction was carried out using a modified Hara and Radin method (Hara and Radin, 1978). Glyceryl triheptadecanoate (17:0) was added as an internal standard during lipid extraction. An equal volume of acid-washed glass beads (0.5 mm) was added to each sample and vortexed vigorously. For phase separation, water: isopropanol: hexane (1:4:6 v/v/v) were added to the sample. The sample was vortexed well and allowed to stand for a few minutes. Aqueous sodium sulfate solution (2.5 ml of 6.6 g anhydrous sodium sulfate in 100 mL) was added and vortexed vigorously, followed by centrifugation for 5 min at 5000 x *g* at room temperature. The upper hexane phase was carefully transferred to another pre-weighed glass tube and dried under nitrogen gas. The total lipid was quantified (mg/g of fresh tissue), resuspended in one mL hexane, and kept at -20°C for further analysis.

### 2.9 Triacylglycerol separation and quantification

TAGs were separated from the total lipid extracts by TLC on Silica Gel 60 plates (Whatman). Lipid extracts were spotted on the TLC plate and developed in a solvent tank containing hexane: diethyl ether: glacial acetic acid (70:30:1, v/v/v). TAGs were visualized by brief exposure to iodine vapor. For charring, plates were sprayed with a solution of 10% (v/v) H_2_SO_4_ in methanol and heated until spots appeared. TAGs were scratched from the TLC plate with a razor and were extracted from the silica plates using the lipid extraction procedure described previously. Lipids were then quantified gravimetrically and processed for composition analyses by gas chromatography (GC).

### 2.10 Determination of TAG fatty acid composition

The fatty acid content and composition of transformed yeast strains were determined by gas chromatography flame ionization detector (GC-FID) (Browse et al., 1986). Briefly, 100 µL of TAG extract was dried under liquid nitrogen and 2 mL of hexane was added. For transmethylation, 200 µL of KOH/MeOH (2M) was added and vortexed for 2 min. To acidify (~ pH 3-4), 400 µL of 2M HCl was added to the mixture. After adding 2 mL of hexane, the sample was centrifuged for 5 min at 3000 x *g* and the upper hexane phase was recovered into a new test tube and dried under nitrogen gas. The dried fatty acid methyl ester was resuspended in 1 mL hexane for analysis by GC-FID (Varian) (Han and Xianlin, 2010). A capillary column (DB-23; 30 m x 0.32 mm I.D., 0.25 µm) was used in the chromatography with the helium carrier gas (flow rate of 1.5 mL/min). The GC-FID column was equilibrated with 2 µL hexane (blank) injection before sample injection. The sample was injected for three min at a temperature of 150°C. Then samples were separated at 150°C for three minutes followed by 150-240°C at 6°C/min, where the detection temperature was 300°C. Each sample was analyzed in triplicate.

Fatty acids from extracted total lipids were converted to fatty acid methyl esters (FAMEs) by heating the samples with two mL of 1N methanolic HCl for 2 h at 85°C in a water bath. Samples were cooled after the transesterification reaction and FAMEs were extracted into the organic phase after vortexing with 1 mL aqueous 0.88% KCl and 1 mL hexane followed by centrifugation for 5 min at 5000 x *g*. The upper hexane phase containing FAMEs was collected into a new test tube, dried under nitrogen gas at 40°C, then resuspended in 100 µL of hexane. One µL of FAME sample was injected into the column for separation and detection by GC-FID. The retention time for each fatty acid was determined by comparison of coelution times of components of standard FAME mixes (Supelco, USA). Fatty acids were quantified relative to known quantities of triheptadecanoin (tri-17:0) internal standard added to each sample prior to lipid extraction.

### 2.11 Nile Red staining and visualization of neutral lipids

Transgenic yeast cells were harvested by centrifugation at 1300 x *g* for 10 min at room temperature, then washed twice each with 1X PBS and sterile distilled water. For sample preparation, 2 µL of Nile Red solution (0.8 mg/mL stock in methanol) was added to 200 µL of yeast cell suspension (1:100 ratio of Nile Red and yeast cells) and incubated at room temperature for 20 min. Portions of the stained cell suspensions were placed on glass slides with coverslips. Agroinfiltrated leaf discs were collected and placed in 50-mL plastic tubes containing 4% paraformaldehyde in 1X PBS on a rotational shaker at 75 rpm for 1 h. Leaf discs were washed three times with 1X PBS and stained with 4 µg/mL Nile Red (4 mg/mL stock in DMSO) in 1X PBS for 15 min at room temperature in a rotational shaker at 100 rpm in the dark. Stained leaf samples were washed three times with 1X PBS and once with ddH_2_O, then mounted on glass slides with coverslips. The microslides containing yeast cells or leaf discs were immediately observed using a Leica TCS SP8 confocal fluorescence microscope with an excitation wavelength of 488 nm and emission at 600-650 nm and 560-620 nm, respectively (n = 3). The total number of LDs was counted for each experiment by ImageJ software. At least three panels from each biological replicate, for a total of nine panels, were analyzed to quantify the LDs. Data were represented as mean ± SD.

### 2.12 Statistical analysis

Statistical analysis was conducted using Minitab statistical software. Data were expressed as their mean value and standard deviation (SD). Student’s paired t-test, and/or one-way ANOVA was used to determine significant differences between the data sets. Pairwise comparisons were made using Tukey’s post-test. The level of significance (p-value) for each analysis is mentioned in the text.

## 3 Results and Discussion

### 3.1 Putative *Pa*DGAT1, *Pa*DGAT2, and *Pa*PDAT1 contain conserved motifs and sequence similarity with plant orthologs

**Figure 1:**
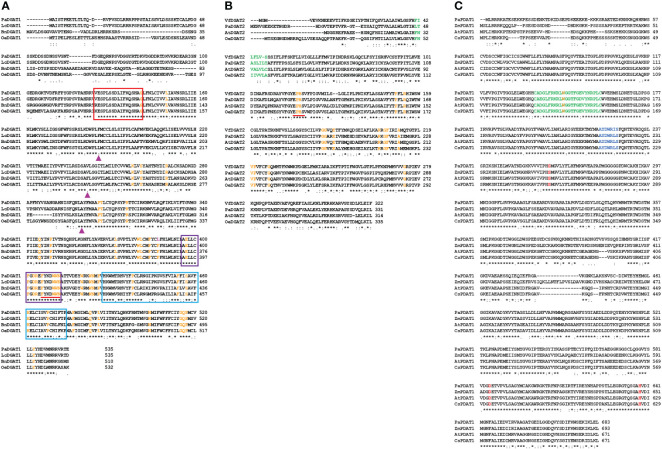
Sequence alignment of *Pa*DGAT1, *Pa*DGAT2, and *Pa*PDAT1 protein sequences to orthologs from different plant species. Orthologous sequences were collected from NCBI and subjected to sequence alignment using ClustalW online tool. Multiple sequence alignment and prediction of conserved motifs and amino acids in plant DGAT1 **(A)**, and DGAT2 **(B)** and PDAT1 **(C)** protein sequences are shown. The binding sites for acyl-CoA (Red), fatty acid (Purple) and DAG (Blue) conserved motifs of DGAT1 are shown in colored boxes. The ‘FYxDWWN’ domain is shown as underlined in red. The conserved ‘L-V-V’ residues among the high TAG plant groups is replaced by ‘F-V-A’ in *Pa*DGAT1 at positions 186, 242, and 300, respectively (▲). The ‘EPHS’ DAG-binding motif in DGAT2 orthologs is shown as underlined in red. The conserved amino acids identified by Cao. H., 2011 in DGAT1 and DGAT2 orthologs are shown in orange color and the substitution (F→L) in *Pa*DGAT1 at position 387 is shown in pink. The lid region, salt bridge and ‘S-D-H’ catalytic triad among the PDAT1 orthologs is shown in green, blue and red color, respectively. Positions having identical (the same) amino acids are indicated by *.

To gain insight into the structural and sequence features of avocado DGAT1, DGAT2, and PDATI, we conducted comprehensive *in silico* analyses using various bioinformatic tools. The predicted polypeptides of *Pa*DGAT1, *Pa*DGAT2, and *Pa*PDAT1 have 535, 331, and 682 amino acid residues, respectively (**Table 1**). The multiple sequence alignment comparisons of *Pa*DGAT1, *Pa*DGAT2, and *Pa*PDAT1 with other related classes of eukaryotic acyltransferases revealed high degree of sequence similarity (**Supplementary Figure 1**) and in some cases, conserved predicted and/or functionally characterized motifs (**Figure 1**). *Pa*DGAT1 is 95.5% similar to the putative DGAT1 from another Lauraceae species, Chinese spicebush (*Lindera communis*) (Dong et al., 2014), and 66% and 68% identical to biochemically characterized orthologs from olive (*Olea europaea*, *Oe*DGAT1) and canola (*Brassica napus*, *Bn*DGAT1), respectively (**Supplementary Figure 1D**). Although relatively few catalytic domains have been experimentally confirmed in DGATs, multiple sequence alignment of several plant DGAT1 protein sequences (including putative *Pa*DGAT1) reveals a set of predicted functional motifs, including putative binding sites for acyl-CoA, fatty acid, and DAG (**Figure 1A**), which were initially identified in *Arabidopsis* DGAT1 (Hobbs et al., 1999; Jako et al., 2001) and *T. majus* DGAT1 (Xu et al., 2008). A known *C*-terminal ER retrieval motif (Shockey et al., 2006) was also identified in the *C*-terminal region of *Pa*DGAT1. Previously, 55 DGAT1 sequences from plants, animals, and fungi were shown to contain 41 conserved amino acids (Cao, 2011), all of which were also retained in *Pa*DGAT1.

*Pa*DGAT2 also showed high similarity with biochemically characterized *Oe*DGAT2 (62.5%), *At*DGAT2 (57.8%), and *Vf*DGAT2 (58.6%) (**Figure 1B**; **Supplementary Figure 1E**). Like other plant DGAT2 sequences analyzed previously (Cao, 2011), *Pa*DGAT2 retained 16 conserved amino acids. Most of the conserved residues in both DGAT families are clustered in the *C*-terminal regions, while the *N*-termini showed greater degrees of variation. Interestingly, as a basal angiosperm, the evolutionary relationships between avocado proteins and other higher plant orthologs can be difficult to predict; in this case, *Pa*DGAT1 and *Pa*DGAT2 were most closely related to orthologs from the monocot species rice (*Oryza sativa*) (**Supplementary Figure 1A**) and maize (*Zea mays*) (**Supplementary Figure 1B**), respectively. The DAG-binding ‘HPHG’ motif first identified in murine DGAT2 (Stone et al., 2006) is replaced with ‘EPHS’ in *Pa*DGAT2 and other plant DGAT2s (**Figure 1B**) (Jeppson et al., 2020). Similarly, PDAT1 sequence alignment showed that avocado PDAT1 is more similar to *Zm*PDAT1 from monocot maize (78.5%) compared to the orthologs from the eudicots *Arabidopsis* (AtPDAT1; 75.2%) and *Cannabis sativa* (*Cs*PDAT1; 74.1%) (**Figure 1C**; **Supplementary Figure 1F**). The PDAT1 *N*-terminal region is more variable, while the *C*-terminal regions are more highly conserved. The lid region, salt bridge, and S-D-H catalytic triad previously shown in *At*PDAT1 are also conserved in all four sequences analyzed here, including *Pa*PDAT1 (**Figure 1C**) (Falarz et al., 2020). The structurally important methionine residue found near the catalytic triad serine is also conserved in *Pa*PDAT1 at position 263 (Falarz et al., 2020).

Membrane topology analyses for the avocado enzymes by TMHMM and Phobius suggested odd numbers of TMDs for both DGATs (**Supplementary Figure 2**). Previous studies demonstrated both even and odd number of TMDs for structurally related plant DGATs (Routaboul et al., 1999; Shockey et al., 2006; Wang et al., 2006). Only one TMD was predicted for *Pa*PDAT1 by both the prediction tools (**Supplementary Figure 2**), consistent with previous functional studies of yeast PDAT (Ghosal et al., 2007). The full summary of *in silico* analytics for avocado DGAT1, DGAT2, and PDAT1 is shown in **Table 1**. We further predicted a structurally disordered region in the *N*-terminal region of *Pa*DGAT1, upstream of predicted TMD1 (**Supplementary Figure 3**). Similar unstructured protein domains, called intrinsically disordered regions (IDRs) (Dyson and Wright, 2005), were also demonstrated in the *N*-terminal cytosolic domain of *Bn*DGAT1 and were implicated in oleoyl-CoA binding (Panigrahi et al., 2018). The IDR of *Bn*DGAT1was shown to undergo secondary structure changes upon ligand binding (Panigrahi et al., 2018) and is responsible for self-dimerization (Caldo et al., 2017). This plant DGAT1 domain also binds PA, a precursor of the DAG substrate and another key regulatory metabolite, leading to upregulation of DGAT activity (Caldo et al., 2017). Hence, it is possible that such regulatory features are intrinsic in *Pa*DGAT1 as well. Future work is necessary to confirm these various structural features and determine their functional role.

### 3.2 *Pa*DGAT1 and *Pa*PDAT1 showed structural conservation of active site residues

**Figure 2:**
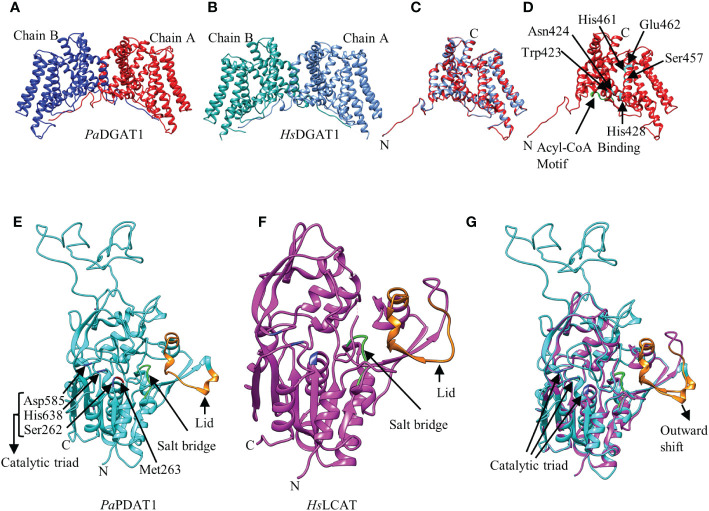
Predicted three-dimensional (3D) structures of *Pa*DGAT1 and *Pa*PDAT1 using SWISS-MODEL interactive workspace. The solved model for human DGAT1 (*Hs*DGAT1; PDB ID: 6VP0) and lecithin:cholesterol acyltransferase (*Hs*LCAT; PDB ID: 5txf) were used to predict the structures of *Pa*DGAT1 and *Pa*PDAT1, respectively. The dimeric structures of *Pa*DGAT1 **(A)** and *Hs*DGAT1 **(B)** are shown followed by structure alignment using chain A **(C)** of both. The predicted acyl-CoA binding motif is shown in green and the substrate binding pocket residues are indicated by amino acid three letter code followed by their respective positions **(D)**. Similarly, the 3D structures of *Pa*PDAT1 **(E)**, *Hs*LCAT **(F)** and their structure alignment **(G)** are shown. The salt bridge and the lid are shown in green and orange, respectively. Predicted 3D model for *Pa*DGAT2 is provided as **Supplementary Figure 4** and the structural assessment parameters for all the three models are provided in **Supplementary Figure 5**.

The 3D structure of the three avocado acyltransferases were predicted by SWISS-MODEL interactive workspace using closest available template (**Figure 2**; **Supplementary Figure 4**). The quality of predicted models based on structural assessment parameters are provided in **Supplementary Figure 5**. *Pa*DGAT1 was predicted using the recently solved 3D structure of human DGAT1 (*Hs*DGAT1, model 6VP0) as a template (**Figures 2A, B**) (Wang et al., 2020). The predicted structure was obtained as a dimer where the *N*-termini of each monomer interact with each other (**Figure 2B**). Structural comparison of the monomers showed that the universally conserved histidine residue (His461) in the MBOAT family proteins is also conserved in *Pa*DGAT1 (**Supplementary Figure 6A**) (Wang et al., 2020). An assessment of the predicted acyl-CoA binding site based on the information from *Hs*DGAT1 showed that the previously predicted loop region FYxDWWN domain is also conserved in *Pa*DGAT1 (**Figure 1A**; **Supplementary Figure 6A**) (Guo et al., 2001; Wang et al., 2020). The Gln465 residue in *Hs*DGAT1 that stabilizes the position of the acyl-CoA thioester for attack by DAG is also conserved in *Pa*DGAT1 at Gln516. The substrate binding pocket residues in *Hs*DGAT1 (Trp377, Asn378, His382, Ser411, His415, Glu416) are also conserved in *Pa*DGAT1 (Trp423, Asn424, His428, Ser457, His461, and Glu462, respectively) (**Figure 2D**; **Supplementary Figure 6A**). The overall structural similarities between *Pa*DGAT1 and *Hs*DGAT1 support the likelihood that the avocado enzyme will effectively utilize oleic acid-containing substrates.

Prediction of the *Pa*DGAT2 3D structure by SWISS-MODEL interactive workspace identified bacterial enzyme *Thermotoga maritima* 1-acyl-*sn*-glycerol-3-phosphate acyltransferase (PlsC) as the best template (PDB ID: 5kym) (**Supplementary Figures 4A, B**). However, the *N*-terminal region (residues 1-93) could not be reliably predicted and thus was not included in the figures shown here. Further structural alignment showed only 15.15% identity (**Supplementary Figures 4C**, **5B**). The catalytic domain ‘HX_4_D’ identified in the PlsC structurally aligned with the ‘PHSVLP’ domain in the *Pa*DGAT2 structure (**Supplementary Figure 4C**) (Robertson et al., 2017), which overlaps with the ‘EPHS’ DAG binding domain (**Supplementary Figure 6B**). Furthermore, analysis of the putative active site and the acyl chain specificity tunnel showed very little similarity and the broad binding tunnel is masked by a 33-residue loop region (positions 241-273) (**Supplementary Figure 4C**). This degree of uncertainty precludes the identification of most of the important domains and key catalytic residues with confidence.

For prediction of the *Pa*PDAT1 3D structure, the human lecithin:cholesterol acyltransferase (*Hs*LCAT, PDB ID: 5txf) was used as template (Glukhova et al., 2015) and the predicted model includes residues 113-659, based on sequence similarities (**Figures 2E, F**). Overall, the structure alignment showed 21.09% identity (**Supplementary Figure 6C**). The lid region, salt bridge, and catalytic triad are shown in orange, green and blue, respectively (**Figure 2E**). The dynamic lid region, previously shown to play a role in controlling access of specific substrates to the active site (Manthei et al., 2017; Falarz et al., 2020) showed variation between the two structures (**Figure 2G**; **Supplementary Figure 6C**). However, such divergent structural variation among plant PDAT1s is common and likely contributes to variation in substrate specificities (Falarz et al., 2020). We hypothesize that the outward shifting of the lid in *Pa*PDAT1 (**Figure 2G**) may facilitate PC entry to the catalytic site. Moreover, the salt bridge, which is responsible for conformational specificity of the active site and substrate recognition, is structurally conserved in *Pa*PDAT1 (Donald et al., 2011; Falarz et al., 2020). Among the S-D-H catalytic triad (Ser262, Asp585, His638) present in avocado PDAT1, the serine residue showed structural variation compared to the human LCAT (**Supplementary Figure 6C**). However, the functionally important Met263 adjacent to the Ser262 in the catalytic triad is structurally conserved (**Figure 2E**; **Supplementary Figure 6C**), similar to that shown in predicted *Arabidopsis* PDAT1 structure (Falarz et al., 2020). Hence, based on our structural analyses, we predict that *Pa*PDAT1 is a functional enzyme with different substrate preference than *Hs*LCAT.

### 3.3 *Pa*DGAT1 activity complements fatty acid lipotoxicity and TAG deficiency phenotypes in TAG-deficient yeast

**Figure 3:**
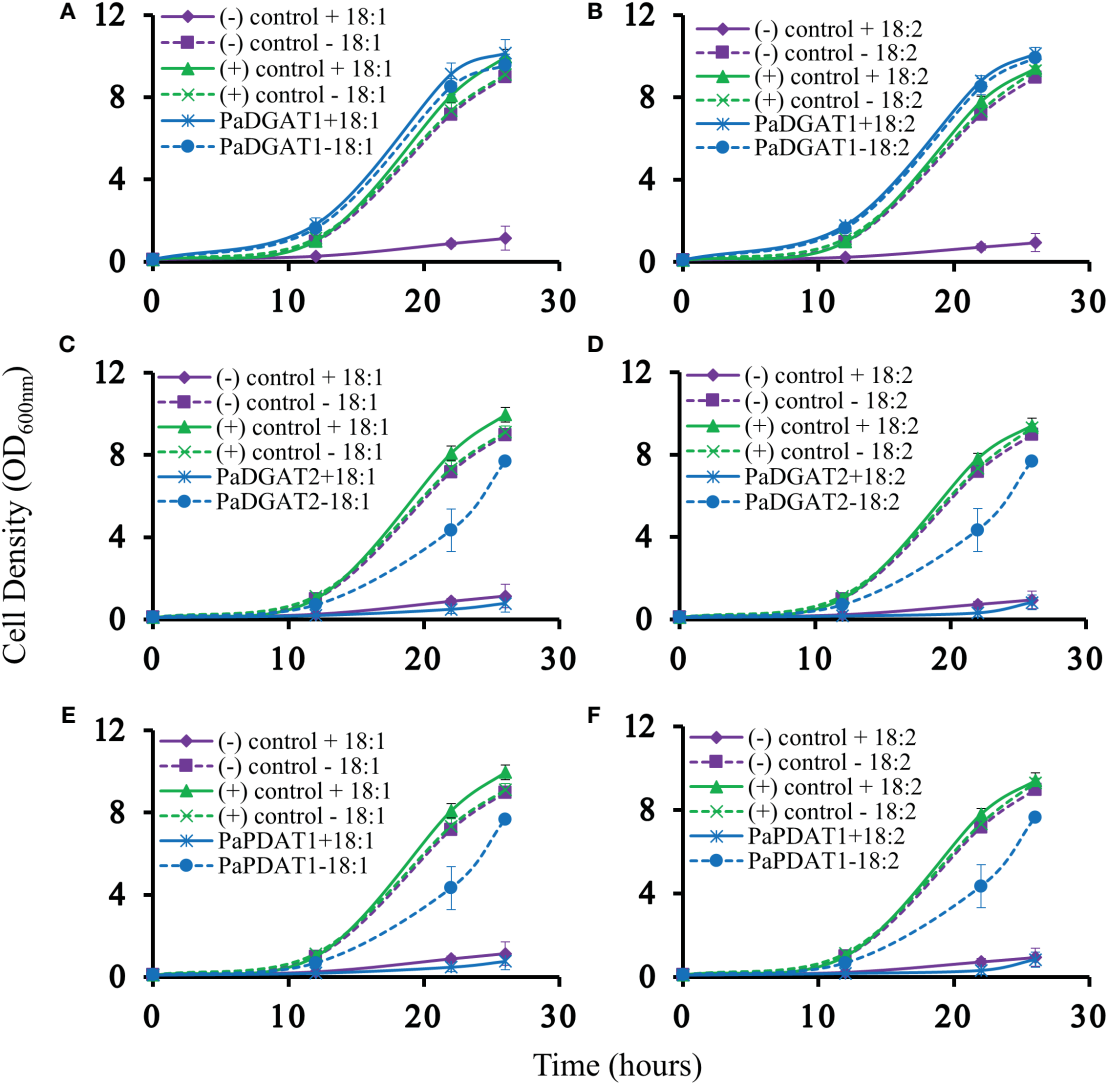
Lipotoxicity rescue complementation of H1246 yeast strain by *Pa*DGAT1, *Pa*DGAT2, and *Pa*PDAT1. Transformed TAG-deficient yeast strain expressing either *PaDGAT1*, *PaDGAT2*, or *PaPDAT1*, along with negative (H1246 containing empty pESC-URA vector) and positive (H1246 transformed with *VfDGAT1*) controls were challenged with 1 mM free oleic acid (18:1) (**A, C**, and **E,** respectively) and 1 mM free linoleic acid (18:2) (**B, D**, and **F**, respectively) in the growth media. Lipotoxicity assays were conducted as an indirect means to assess TAG biosynthetic competency of each enzyme. Only *Pa*DGAT1 complementation rescued H1246 yeast strain in the presence of 18:1 or 18:2, as indicated by growth curves similar to those of positive control.

Our previous bioinformatic and transcriptomic analyses suggested that TAG biosynthesis in avocado mesocarp is likely controlled in large part by *Pa*DGAT1, *Pa*DGAT2, and *Pa*PDAT1 activities (Kilaru et al., 2015), so we directly tested for TAG synthesis by avocado acyltransferase overexpression in yeast. We used the H1246 mutant strain of *S. cerevisiae* that lacks all four acyltransferases (Are1p and Are2p: sterol O-acyltransferases 1 and 2, Dga1p: diacylglycerol acyltransferase, and Lro1p: ortholog of yeast PDAT1) necessary for TAG and stearyl ester synthesis and thus is devoid of neutral lipids (Sandager et al., 2002). H1246 is sensitive to free fatty acids (FFAs) in the growth media, thus making it an ideal host for testing DGATs, PDATs, and other TAG biosynthetic genes for functional activity that will restore TAG production and rescue the cells from the FFA lipotoxic effects (Siloto et al., 2009; Pan et al., 2013). H1246 was transformed with expression plasmids bearing either *PaDGAT1*, *PaDGAT2*, or *PaPDAT1* and grown in the presence of either oleic acid or polyunsaturated (18:2) FFAs. Culture growth rates were compared to that of positive (*Vf*DGAT1) and negative (pESC-URA empty vector) controls (Shockey et al., 2006), with various time points sampled over a total of 26 hours (**Figure 3**). Growth curves of each strain were also compared to equivalent cultures grown in liquid media lacking fatty acids. Both oleic and linoleic acid completely inhibited growth of the negative control. On the other hand, growth of the positive control and the *Pa*DGAT1 strains were nearly identical in the presence and absence of 18:1 or 18:2 fatty acids (**Figure 3**). The ability to utilize FFAs from the media and prevent lipotoxic effects confirmed functional activity for *Pa*DGAT1 (**Figures 3A, B**). *PaDGAT2* however, was unable to complement H1246 in its native form (data not shown) or when expressed from a yeast codon-optimized synthetic gene (**Figures 3C, D**). *Pa*PDAT1 also failed to overcome the lipotoxic effect when expressed in H1246 yeast strain (**Figures 3E, F**). Though not a direct assessment of substrate specificity, the FFA lipotoxicity assay can provide some insight to enzyme selectivity. *Arabidopsis* DGAT1 successfully rescued H1246 when the growth media was supplemented with oleic acid (a major component of polar and neutral lipids in *Arabidopsis*) but not with medium-chain saturated fatty acids that are not typically produced in this plant (Iskandarov et al., 2017). Similarly, human DGAT2 reverted the lipotoxicity in H1246 in the presence of unsaturated fatty acids (Garbarino and Sturley, 2009). The results shown here establish that *Pa*DGAT1 is an active acyltransferase and may be a factor in the enrichment of oleic and linoleic acids in avocado TAG.

Previous studies indicate that DGAT2 enzymes may require as-yet unknown additional factors for optimal expression and *in vitro* enzyme assay conditions (Stone et al., 2004; Yen et al., 2008). Likewise, other studies suggest that low steady-state protein levels for transgenic DGAT2s may contribute to this phenomenon when expressed in yeast and other heterologous cell types. *Arabidopsis* DGAT2 (Aymé et al., 2014; Chen et al., 2015) and fungal *Umbelopsis ramanniana* DGAT2 (Lardizabal et al., 2008) showed almost no DGAT activity in yeast and/or were undetectable by western blotting. Codon optimization is a frequent adaptation used to overcome this issue, with mixed results. Optimized *At*DGAT2 gained orders of magnitude of protein stability relative to its native counterpart (Aymé et al., 2014), but optimization did not improve *Pa*DGAT2 expression enough to rescue the lipotoxic phenotype in H1246 yeast. The cause of these negative results is not clear, but it is also possible that *Pa*DGAT2 may play a highly specialized role in a limited number of cell or tissue types. We also questioned whether a mutation in *PaDGAT2* could have resulted in a loss, or reduction, of activity or protein stability. Previous studies showed that evolutionary drift at phenylalanine 469 in maize DGAT1-2 affected its activity in maize (Zheng et al., 2008). Interestingly, we found that among a large group of plant DGAT2s covering dozens of species, only *Pa*DGAT2 contains a tyrosine at position 314, while all other DGAT2s contain phenylalanine (**Supplementary Figure 7**). We suspect that *Pa*DGAT2 may have undergone a point mutation resulting in a loss of function. Future site-directed mutagenesis studies aimed at replacing Tyr314 with Phe could reveal if a reversion at this residue could favorably alter *Pa*DGAT2 enzyme activity.

### 3.4 *Pa*DGAT1 encodes a protein with TAG biosynthetic activity

**Figure 4:**
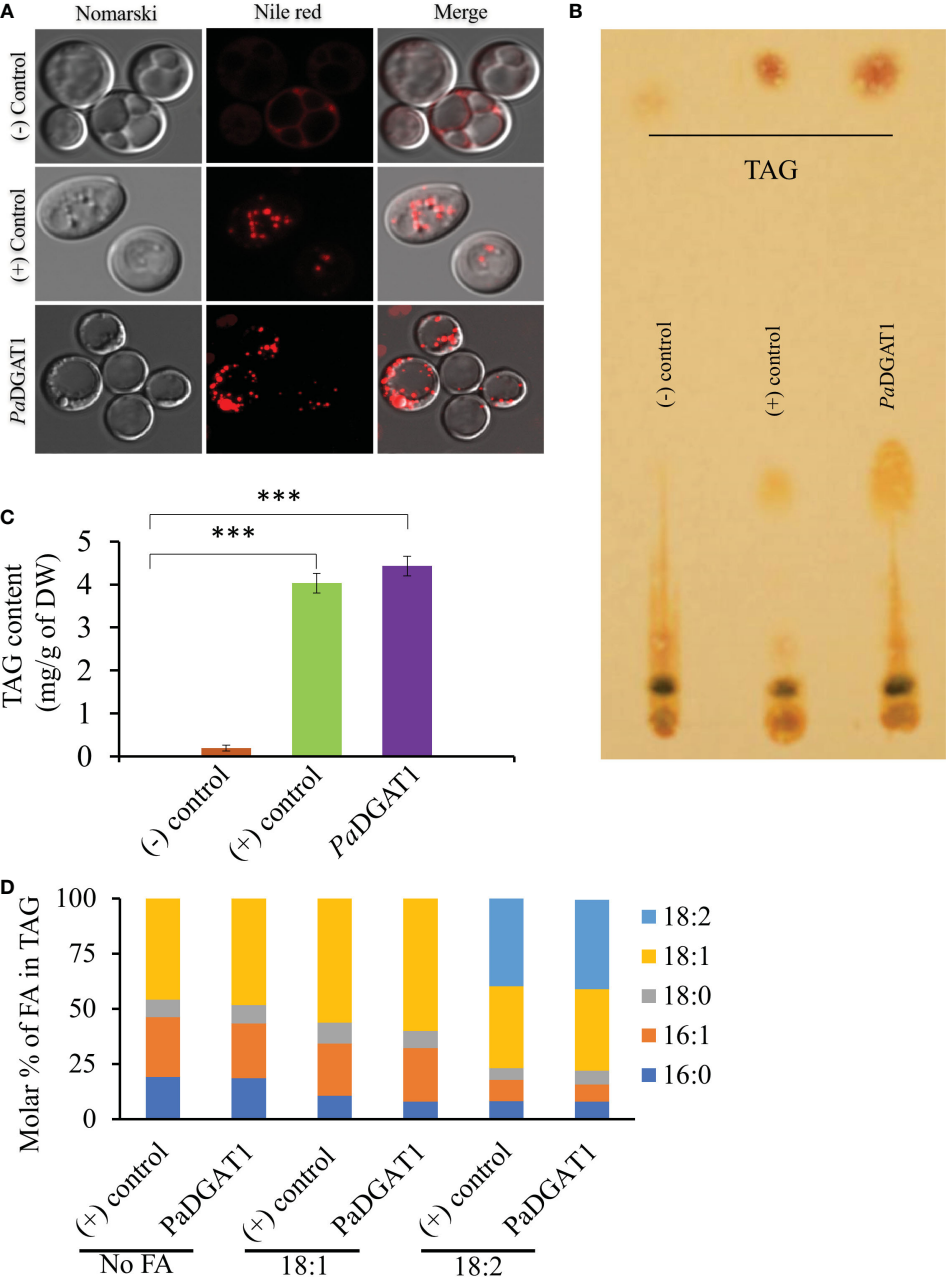
Observation of newly synthesized lipid droplets (LD) in complemented yeast strain and determination of TAG content and composition. TAG-deficient yeast cells expressing *PaDGAT1* restored LD formation, as visualized by staining the cells with the lipid-specific fluorescent dye Nile Red **(A)**. The neutral lipid deficient quadruple mutant H1246 strain harboring *VfDGAT1* was used as a positive control. The quadruple mutant H1246 strain containing empty plasmid was used as a negative control. Nile Red fluorescence was observed with an excitation of 488 nm and an emission of 600-650 nm wavelength. Lipids were extracted from yeast cultures grown under protein expression inducing conditions for 24 h, then separated by TLC **(B)**. Fatty acid content **(C)** and composition **(D)** were quantified by GC-FID for cultures grown in media containing either no fatty acid supplementation, or 1 mM oleic acid (18:1) or linoleic acid (18:2). Experiments were done in triplicate and the data are mean ± SD, n=3. ***P<0.001.

The survival and growth of *PaDGAT1*-expressing yeast strain H1246 in the presence of FFAs should correlate with accumulation of neutral lipids, including TAG, which are stored in LDs. Microscopic analysis of H1246 cells expressing *Pa*DGAT1 or *Vf*DGAT1 (positive control) confirmed production of LDs. On the contrary, negative control mutant cells failed to accumulate any neutral lipids (**Figure 4A**). TLC analyses of total lipids extracted from these cells confirmed restoration of TAG biosynthesis in both the positive control and *Pa*DGAT1 strains, but not the negative control (**Figures 4B, C**). These results directly confirm that *Pa*DGAT1 encodes a protein with TAG biosynthetic activity. Total cellular fatty acid composition was analyzed in transgenic yeast cells either grown without fatty acid supplementation or with either 18:1 or 18:2 added to the media. After 48 hours, yeast cells were harvested, and fatty acid composition was analyzed from total TAG content. In feeding studies, 16:0, 16:1, and 18:0 were produced by endogenous *de novo* fatty acid synthesis. Oleic acid can be produced directly by the yeast as well (in media lacking fatty acid supplementation) or may constitute a mixture of endogenous and exogenous oleate, in cultures fed with this fatty acid. Yeast does not have any intrinsic 18:2 biosynthetic capacity, therefore all 18:2 quantified from yeast comes only from the media supplement. In the absence of a fatty acid supplement, the fatty acid composition which is incorporated by *Pa*DGAT1 strains is dominated by 18:1, making up ~46% of the total fatty acids. Oleate-supplemented *Pa*DGAT1 cells contain ~70% 18:1. Interestingly, while linoleate supplementation increases 18:2 content to ~40%, *Pa*DGAT1 strains grown under this condition still retained nearly equivalent amount of 18:1, in this case drawn entirely from the endogenous fatty acid pool (**Figure 4D**).

### 3.5 *Pa*DGAT1 preferentially incorporates oleic acid (18:1) during TAG accumulation

**Figure 5:**
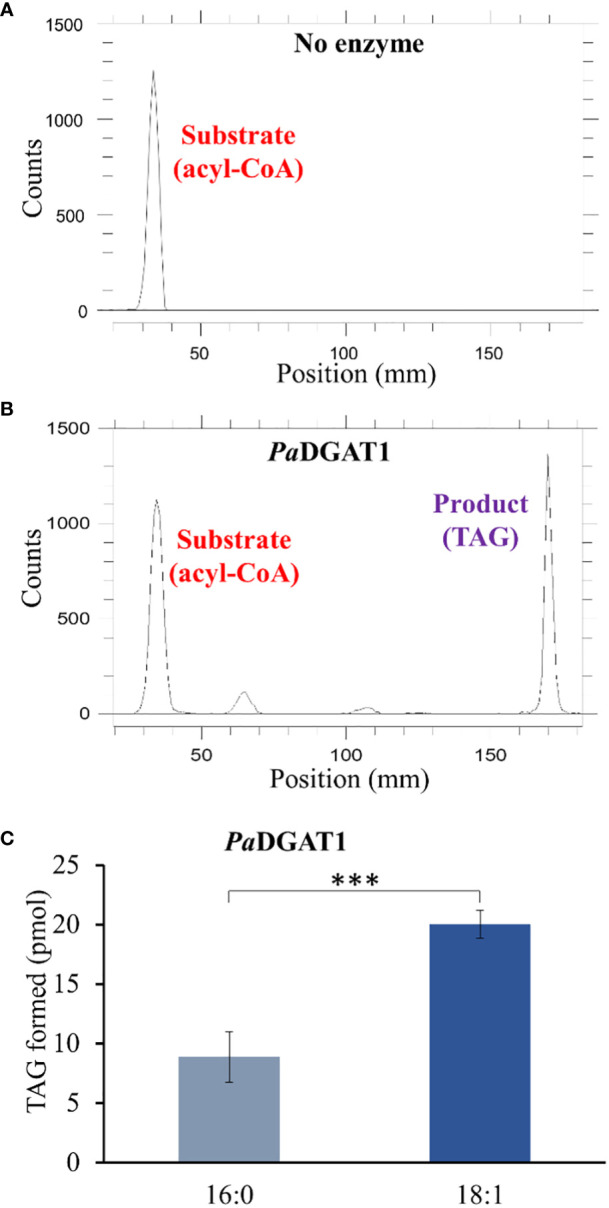
*In vitro* enzyme activity and specificity assays for yeast expressing PaDGAT1. PaDGAT1 with an N-terminal HA epitope tag was expressed in the H1246 yeast strain and was induced for 24 h. Microsomal fractions were prepared and incubated with radiolabeled fatty acyl-CoA and DAG. Saturated (palmitoyl-CoA, 16:0-CoA) and monounsaturated (oleoyl-CoA, 18:1-CoA) acyl donor substrates were tested. Representative chromatogram generated by Radio-TLC imaging scanner for negative control with only acyl-CoA substrate and no enzyme **A)**, and PaDGAT1 microsomes incubated with 16:0 or 18:0 acyl-CoA and diolein that resulted in TAG synthesis **(B)**. Quantification of TAG product peaks revealed that PaDGAT1 prefers 18:1-CoA over 16:0-CoA **(C)**. ***P<0.001.

The preferential accumulation of oleic acid in TAGs from transgenic *PaDGAT1*-expressing yeast was intriguing. To investigate the biochemical properties of this enzyme more directly, we performed *in vitro* enzyme assays using radiolabeled 16:0- or 18:1-CoAs as substrates (**Figures 5A, B**). Our results suggest that *Pa*DGAT1 utilizes both 16:0 and 18:1, but it showed a two-fold higher preference for 18:1-CoA than 16:0-CoA **(** **Figure 5C**). The substrate preference for DGAT1 in plants differs depending on the species and tissue types and on the availability of the substrates in that species (Shockey et al., 2006). Plant DGAT1 has a wide range of substrate specificities from medium to long-chain, and saturated, monounsaturated, or polyunsaturated fatty acids. Ancestral maize DGAT1-2 had a high preference for oleic acid but partially lost specificity and enzyme efficiency upon loss of phenylalanine-469 during the breeding and domestication of this crop (Zheng et al., 2008). *Brassica napus* DGAT1 also had a higher preference for 18:1 compared to 16:0. The high preference of *Bn*DGAT1 for 18:1 positively correlates with the seed fatty acid composition (Aznar-Moreno et al., 2015). Similarly, in avocado, the preference of DGAT1 towards 18:1 correlates with the fatty acid composition of avocado mesocarp (Kilaru et al., 2015).

### 3.6 Transient expression of *PaDGAT1* and *PaPDAT1* in *N. benthamiana* leaves leads to TAG accumulation

**Figure 6:**
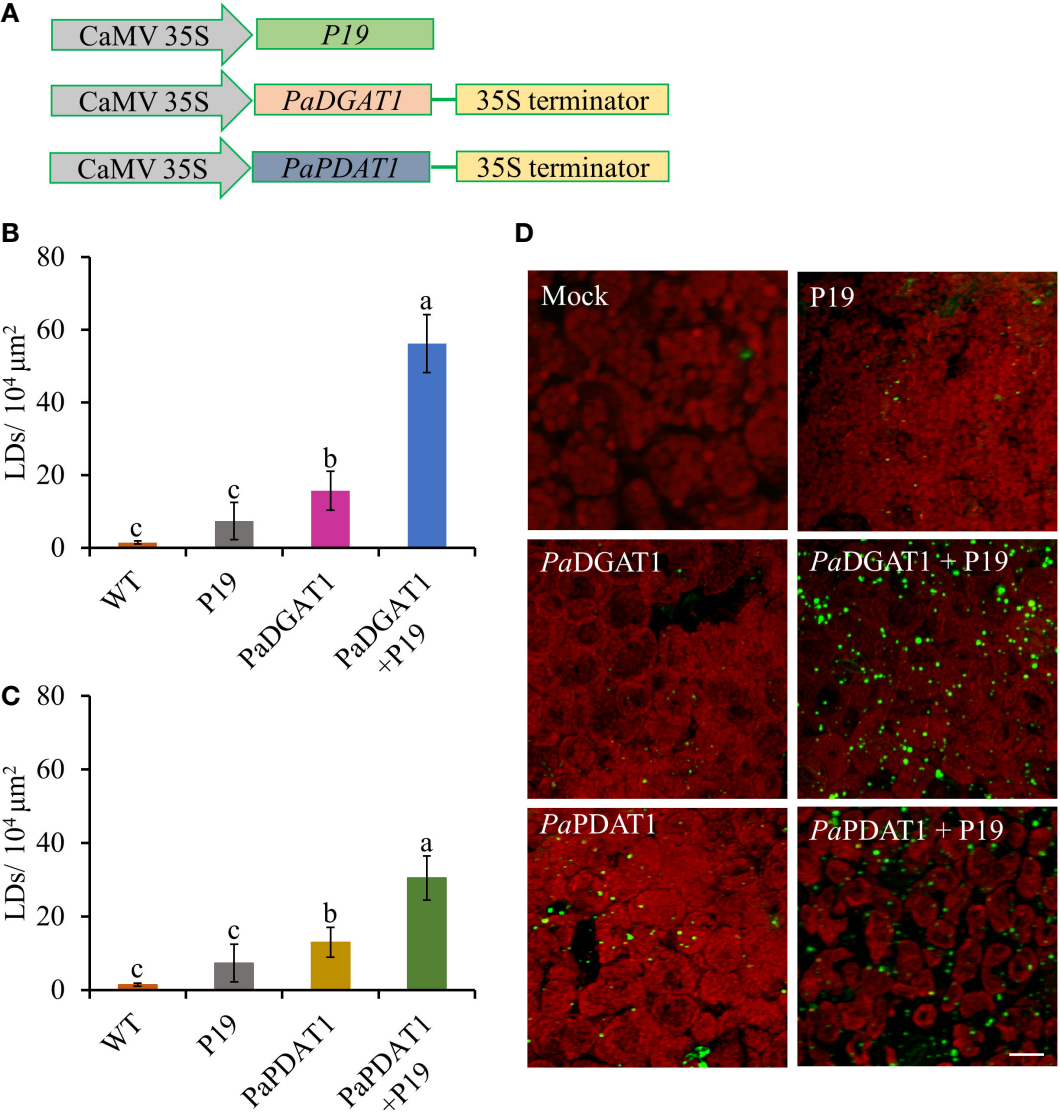
Vector constructs, LD visualization, and quantification of LDs in *N. benthamiana* leaves expressing *Pa*DGAT1 and *Pa*PDAT1. Schematic diagrams for P19, *PaDGAT1*, and *PaPDAT1* vector constructs used for transient *Agrobacterium* transformation of *N. benthamiana* leaves **(A)**. Quantification of LDs in different *N. benthamiana* leaves expressing *PaDGAT1* **(B)** and *PaPDAT1* **(C)**. Data represent mean ± SD of three independent experiments and different letters indicate significant differences (P < 0.05), as determined by ANOVA with Tukey’s post-test. Phenotypic visualization of LDs (in green color) in *N. benthamiana* leaves expressing either *PaDGAT1* or *PaPDAT1* alone or with P19 **(D)**. LDs were stained with Nile Red then visualized by confocal microscopy; leaves with mock infiltration or p19 expression alone are used as negative controls. Scale bar represents 20 µm.

Although our yeast transgene expression studies using *Pa*PDAT1 failed to confirm a functional role in TAG biosynthesis, based on its high expression level in the mesocarp (Kilaru et al., 2015), we still suspected that it might be involved in avocado oil metabolism. Hence, we further assessed *PaDGAT1* and *PaPDAT1* for their functional role through *in planta* expression of these genes in *N. benthamiana* leaves. The ‘benth leaf system’, as it is commonly known, has become a valuable experimental system, due to its speed and ease of use, thus providing a plant cell environment that can be more conducive to studies of plant enzymes (Grimberg et al., 2015; Reynolds et al., 2015a; Delatte et al., 2018). Among many other plant DGATs and other lipid metabolic enzymes, *Arabidopsis* DGAT1 was expressed in this system and dramatically increased the number of LDs produced (Vanhercke et al., 2014; Vanhercke et al., 2017). Co-expression of *Arabidopsis* DGAT1 with the *WRINKLED1* transcription factor increased TAG content in benth leaves up to 15% of dry weight (Vanhercke et al., 2013; Reynolds et al., 2015b). Thus, we transiently expressed *PaDGAT1* and *PaPDAT1* in benth leaves, individually or combined with the viral silencing suppressor protein P19 (Wood et al., 2009), under the control of the *CaMV35S* promoter (**Figure 6A**) and observed accumulated LDs by confocal fluorescence microscopy. We observed that *Pa*DGAT1 produced elevated LD counts compared to wild-type, which provided further evidence for TAG biosynthesis by this enzyme (**Figure 6B**). The number of LDs produced by *Pa*DGAT1 in *N. benthamiana* leaves was almost 10-fold and 2-fold higher than those in mock and P19 negative controls (Students’ T-test, *P*< 0.01 and *P*<0.05, respectively) (**Figure 6C**). The combination of *Pa*DGAT1 with P19 protein had a more robust effect, where the number of LDs increased by 37-fold and 7-fold compared to the wild-type (*P*<0.001) and P19 control (*P*<0.001), respectively (**Figure 6C**). A similar trend was observed when *PaPDAT1* was transiently expressed in benth leaves, where the number of LDs increased by 4-fold and 2-fold compared to wild-type control and *P19* control, respectively. Co-infiltration of *PaPDAT1* with *P19* resulted in an elevation of LDs by 22-fold and 4-fold compared to wild-type (*P*<0.001) and P19 control (*P*<0.001), respectively (**Figure 6D**). The P19 viral silencing suppressor is included here to help suppress transgene silencing, a common problem in both transiently and stably transformed plant cells (Naim et al., 2016). It is not completely clear why *P19* expression alone can increase LD production in benth leaves, but it is possible that P19 protein affected the expression levels of endogenous enzymes to a certain extent. In any case, the increase in LD count induced by *Pa*DGAT1 and *Pa*PDAT1, relative to P19 control confirm acyltransferase activity and strongly suggest that *Pa*DGAT1 and *Pa*PDAT1 are functional and thus, responsible for TAG production in nonseed avocado tissues.

**Figure 7:**
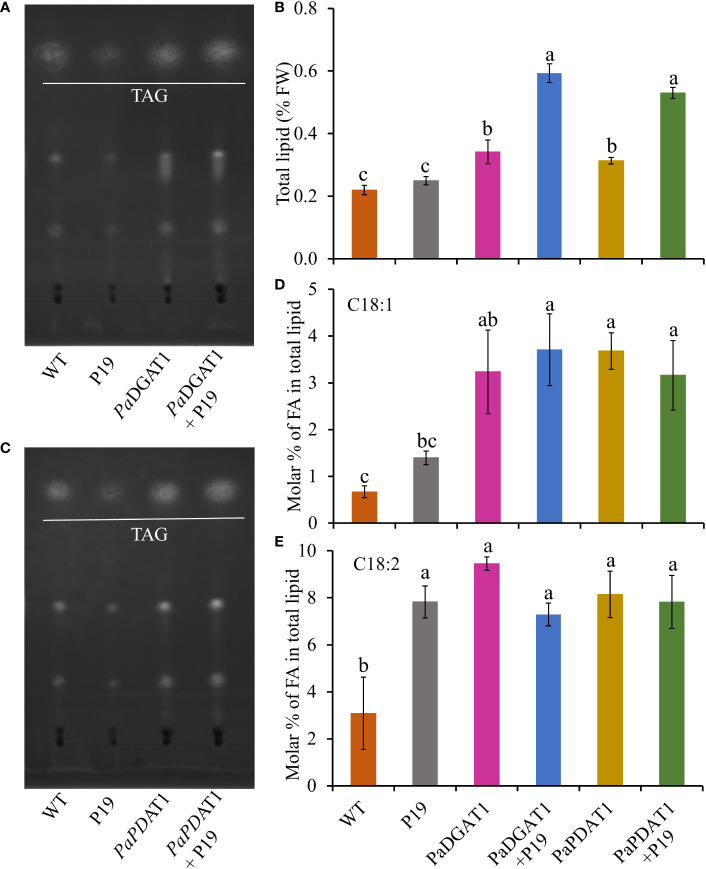
Quantification of total lipid content in *N. benthamiana* leaves expressing *PaDGAT1 or PaPDAT1.* Separation of total lipid by TLC from *N. benthamiana* leaves expressing *PaDGAT1* **(A)** or *PaPDAT1* **(C)**. Quantification of total lipid from benth leaves expressing *PaDGAT1* or *PaPDAT1*, **(B)**. Fatty acid composition of total lipid content was analyzed and quantified by GC-FID in benth leaves expressing *PaDGAT1* or *PaPDAT1* with or without P19 coexpression. The molar percentage composition for C18:1 **(D)** and C18:2 **(E)** is shown. Data represent mean ± SD of three independent experiments and different letters indicate significant differences (P < 0.05), as determined by ANOVA with Tukey’s post-test.

In addition to the observed changes in LD numbers in *N. benthamiana* cells expressing avocado acyltransferases, we directly determined the change in total lipid content in benth leaves expressing *PaDGAT1* or *PaPDAT1.* We extracted total lipid from infiltrated leaves expressing *PaDGAT1, PaPDAT1* or controls and separated them by TLC (**Figures 7A, C**). The expression of *PaDGAT1* significantly increased (>2-fold) total lipid content compared to either wild-type or P19 controls (P<0.01) (**Figure 7B**). Likewise, *PaPDAT1* expression increased total lipid content by ~2-fold (**Figure 7B**). These results indicate that transient overexpression of *PaDGAT1* and *PaPDAT1* increased total lipid content in benth leaves specifically by making more TAGs.

### 3.7 *Pa*DGAT1 and *Pa*PDAT1 show a preference for oleic acid when expressed in *N. benthamiana* leaves

To gain more insight into the substrate specificities of *Pa*DGAT1 and *Pa*PDAT1 expressed in plant vegetative tissues, we examined the fatty acid profile in *N. benthamiana* leaves by GC-FID. The content of 16:0, 16:1, and 18:3 did not change significantly in response to *PaDGAT1* or *PaPDAT1* expression (**Supplementary Table 1**), compared to the wild-type or P19 controls. However, *Pa*DGAT1 activity significantly increased oleic acid content compared to wild-type (*P*<0.05); viral silencing suppressor protein *P19* expression enhanced this effect (vs WT controls, *P*<0.001; vs P19 controls, P<0.05) (**Figure 7D**). *PaPDAT1* produced similar results (**Figure 7D**). Expression of both avocado acyltransferases significantly increased 18:2 levels over wild-type controls, but neither *Pa*DGAT1 nor *Pa*PDAT1 activity elevated linoleic acid content over the P19 background levels (**Figure 7E**).

Our results for *Pa*DGAT1 were consistent with those of Vanhercke et al. (2014), who reported that *Arabidopsis* DGAT1 enhanced oleic acid levels in *N. tabacum* leaves. Given the unsuccessful detection of *Pa*PDAT1 activity in yeast cells, transient expression of this enzyme in *N. benthamiana* leaves provided the first direct evidence of TAG biosynthetic activity and suggested a similar preference for oleic acid-containing substrates as seen with *Pa*DGAT1; further validation by *in vitro* PDAT enzyme activity assays are still warranted. Although *Arabidopsis* PDAT was active with a broad spectrum of substrates containing acyl chains of varying lengths, it showed strong preferences for hydroxy, epoxy, and polyunsaturated fatty acids (Ståhl et al., 2004), suggesting that this class of enzymes may also play a role in membrane surveillance and turnover of oxidized or otherwise damaged fatty acids. For example, *Camelina sativa* PDAT1-A increased polyunsaturated fatty acids (18:2 and 18:3) compared to wild-type control in two independent studies (Marmon et al., 2017; Yuan et al., 2017). Similarly, castor bean *Rc*PDAT1A showed specificity toward the hydroxy fatty acid ricinoleic acid (van Erp et al., 2011). Interestingly, while flax PDAT1 showed strong preference for 18:3, PDAT2 preferred other polyunsaturated fatty acids (Pan et al., 2013). These camelina, castor, and flax results show positive correlation between PDAT activity and the dominant fatty acid present in their respective seed oils. The different substrate preferences by various PDAT paralogs and orthologs indicate its divergent functional role in plants. In the case of avocado, the coordinate evolution towards oleic acid preference by *Pa*DGAT1 and *Pa*PDAT1 strongly suggests a role for both enzymes in controlling the final fatty acid profile of its mesocarp oils.

### 3.8 Transient co-expression of *PaDGAT1* and *PaPDAT1* further improved TAG production in *N. benthamiana* leaves

**Figure 8:**
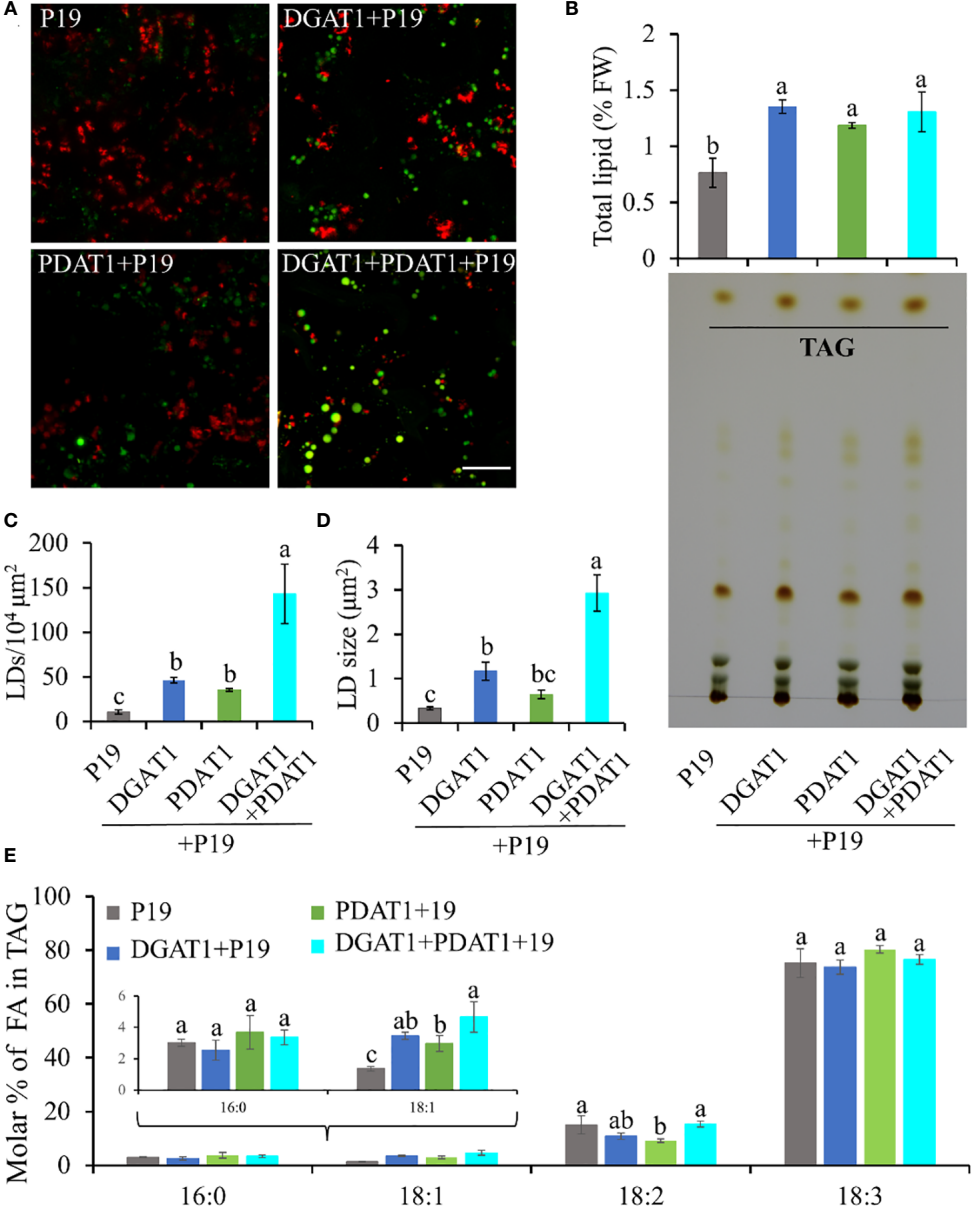
Quantification of LD accumulation and fatty acid profile in TAG in N. benthamiana leaves co-expressing PaDGAT1 and PaPDAT1. Confocal images of the accumulated LDs stained with Nile Red (green) in N. benthamiana leaves expressing control (+P19), DGAT1 (+P19), PDAT1 (+P19), and DGAT1 + PDAT1 (+P19) **(A)**. The scale bar represents 20 μm. Quantification of total lipid extracted from the leaves and separation of TAG by TLC **(B)**. Number of accumulated LDs per unit area **(C)** and their average size (area) **(D)**. Fatty acid profile of extracted TAG from the TLC plates **(E)**. Data represent mean ± SD of three independent experiments and different letters indicate significant differences (P < 0.05), as determined by ANOVA with Tukey’s post-test.

Since both DGAT1 and PDAT1 in avocado are functional in TAG biosynthesis, we hypothesized that the continuous high expression of both genes in mesocarp tissue cooperatively achieves high oleic acid accumulation in TAG. Although DGAT1 and PDAT1 have overlapping functions in at least some seeds (Zhang et al., 2009), their relative contribution to the TAG pool is highly variable and is species-specific (Banaś et al., 2013). Here we assessed the combined effect of *Pa*DGAT1 and *Pa*PDAT1 on TAG biosynthesis in nonseed tissue, which, to our knowledge, has not been explored for other plant species. We co-expressed *PaDGAT1* and *PaPDAT1* along with *P19* in *N. benthamiana* leaves; *P19* expression alone was used as the control. Interestingly, *DGAT1*+*PDAT1*+*P19* co-expression increased the LD accumulation in the leaf tissue by ~3-fold and ~4-fold compared to independent expression of *DGAT1+P19* and *PDAT1+P19*, respectively (**Figures 8A, C**). The increase in LD count was more than additive, i.e., the co-expression resulted in a greater LD accumulation compared to the sum of LDs shown by DGAT1 and PDAT1. The LD accumulation in animals and plants is a dynamic process and may be triggered to maintain energy homeostasis, regulate membrane remodeling and as a response to stress (Chitraju et al., 2017; Pyc et al., 2017; Welte and Gould, 2017; Yang and Benning, 2018; Fernández-Santos et al., 2020). It is possible that the elevated LD count occurs in part due to the stress induced by the *DGAT1*/*PDAT1* co-expression, as a reduction in chlorophyll was noticeable (**Figure 8A**). Co-expression also increased average LD size (area) increase by >2-fold and >4-fold compared to single expression of *DGAT1* and *PDAT1*, respectively (**Figure 8D**), suggesting improved LD production and packaging efficiency. However, there was no significant improvement in the total lipid content in leaves (**Figure 8B**), which might suggest that co-expression did not increase *de novo* fatty acid biosynthesis but contributed to elevated TAG assembly. Previously, an increase in the PC pool upon *At*DGAT1 expression in tobacco leaves was reported (Andrianov et al., 2010; Vanhercke et al., 2013). Similarly, such an increase in PC might be used as a substrate for utilization by *Pa*PDAT1 contributing to TAG assembly. Recently it was shown that *At*DGAT1 and *At*PDAT1 physically interact with each other both *in planta* and in yeast two hybrid system to maximize TAG biosynthesis (Lee and Seo, 2019; Regmi et al., 2020). Possibly, such interaction between *Pa*DGAT1 and *Pa*PDAT1 might result in a rapid carbon flux into TAG in this micro-environment, which further leads to LD size increase. Further experimental verification is needed to confirm avocado DGAT1/PDAT1 interactions.

Since both *Pa*DGAT1 and *Pa*PDAT1 preferred oleic acid as substrate in our previous experiments, we further analyzed the fatty acid profile of TAG to see if there is any further improvement in oleic acid content. TAG was separated from the total lipid pool by TLC (**Figure 8B**), methylated and analyzed by GC-FID. Our results suggest an increase in the proportion of oleic acid in the TAG by >30% and >50% in the *DGAT1*+*PDAT1+P19*-expressed leaves compared to the leaves expressing *DGAT1*+*P19* and *PDAT1*+*P19*, respectively (**Figure 8E**; **Supplementary Table 2**). However, the improvement was statistically significant compared to only the *PDAT1*+*P19*. Nonetheless, the results suggest that the synchronous activity of DGAT1 and PDAT1 in avocado mesocarp is responsible for the high proportion of oleic acid in the synthesized TAG since it was significantly (by ~2.4 fold) increased compared to the P19 control. Interestingly, 18:2 content in the TAG from *DGAT1*+*PDAT1*+*P19-*expressed leaves is similar to that from *P19* control leaves, although it is significantly reduced in *PDAT1*+*P19*-expressed leaves (**Figure 8E**). This suggests that combination of *Pa*DGAT1 and *Pa*PDAT1 is a better strategy to specifically improve oleic acid content without affecting other aspects of fatty acid composition.

## 4 Conclusions

We have identified and functionally characterized *PaDGAT1* and *PaPDAT1* by complementation of yeast quadruple mutant H1246 and/or transient expression in *N. benthamiana* leaves. Heterologous expression and *in vitro* enzyme activity confirmed the diacylglycerol acyltransferase activity of *PaDGAT1* and its preference for oleic acid-containing substrates. Transient expression of *PaDGAT1* and *PaPDAT1* in *N. benthamiana* leaves also revealed the acyltransferase activity and substrate specificity for oleic acid. The present findings suggest that *PaDGAT1* and *PaPDAT1* cooperatively play roles in TAG assembly and provide new insight into the underlying mechanism of lipid biosynthesis in avocado mesocarp. The results obtained from this study will enrich our knowledge towards an understanding of lipid biosynthesis in nonseed tissue and can be translated into other plant species or less complex organisms, such as oleaginous microbes to produce oil enriched in oleic acid.

## Data availability statement

The original contributions presented in the study are publicly available. This data can be found here: GenBank OP727298, OP727299 and OP727300.

## Author contributions

MR, JS, JB and AK designed the research. MR, JB and JS conducted the experiments in yeast and *N. benthamiana*. JB and MR conducted the bioinformatics analyses. JB, MR, JS, and AK conducted the data analysis. JB and MR wrote the manuscript and JS and AK edited the manuscript. All authors contributed to the article and approved the submitted version.

## Supplementary material

Supplementary Figure 1 Phylogenetic analysis and percentage sequence similarities of avocado DGAT1, DGAT2, and PDAT1 to orthologous plant proteins. Phylogenetic analysis using MEGA X software.

Supplementary Figure 2 Predicted avocado DGAT1, DGAT2, and PDAT2 membrane topologies by TMHMM and Phobius.

Supplementary Figure 3 Predicted intrinsically disordered region (IDR) in the *N*-terminal region of *Pa*DGAT1.

Supplementary Figure 4 Predicted three-dimensional (3D) structures of *Pa*DGAT2 by the SWISS-MODEL interactive workspace tools, using the solved or predicted models for *Thermotoga maritima* 1-acyl-sn-glycerol-3-phosphate acyltransferase (PlsC) (PDB Id: 5kym). The 3D structures of *Pa*DGAT2 **(A**) and PlsC **(B)** followed by their structural comparison **(C)** showing the *Pa*DGAT2 in cyan, PlsC in red and Hx4D domain in blue, respectively.

Supplementary Figure 5 The quality of the predicted 3D structures based on the structure assessment. The Ramachandran plot showing the bond angles (Φ and Ψ) for amino acids at each position, the quality of the predicted structure assessed by QMEANDisCo and MolProbity for *Pa*DGAT1 **(A)**, *Pa*DGAT2 **(B)**, and *Pa*PDAT1 **(C)** are shown.

Supplementary Figure 6 RMSD values of structure-based sequence alignments of *Pa*DGAT1, *Pa*DGAT2, and *Pa*PDAT1 with their respective templates.

Supplementary Figure 7 Sequence alignment of *C*-terminal regions of additional plant DGAT2 proteins. The invariant Phe residue that has been changed to a Tyr residue in avocado DGAT2 (shown in red) is boxed.
