# Erratum: Acyl-CoA-dependent and acyl-CoA-independent avocado acyltransferases positively influence oleic acid content in nonseed triacylglycerols

**DOI:** 10.3389/fpls.2023.1158034

**Published:** 2023-02-16

**Authors:** Frontiers Production Office

**Affiliations:** Frontiers Media SA, Lausanne, Switzerland

**Keywords:** triacylglycerol, avocado, nonseed, oleic acid, DGAT1, DGAT2, PDAT1

Due to a production error, we missed implementing change requests to correct typos, formatting, and to use the final version of **Figures 5**, **8**.

**Figure 5:**
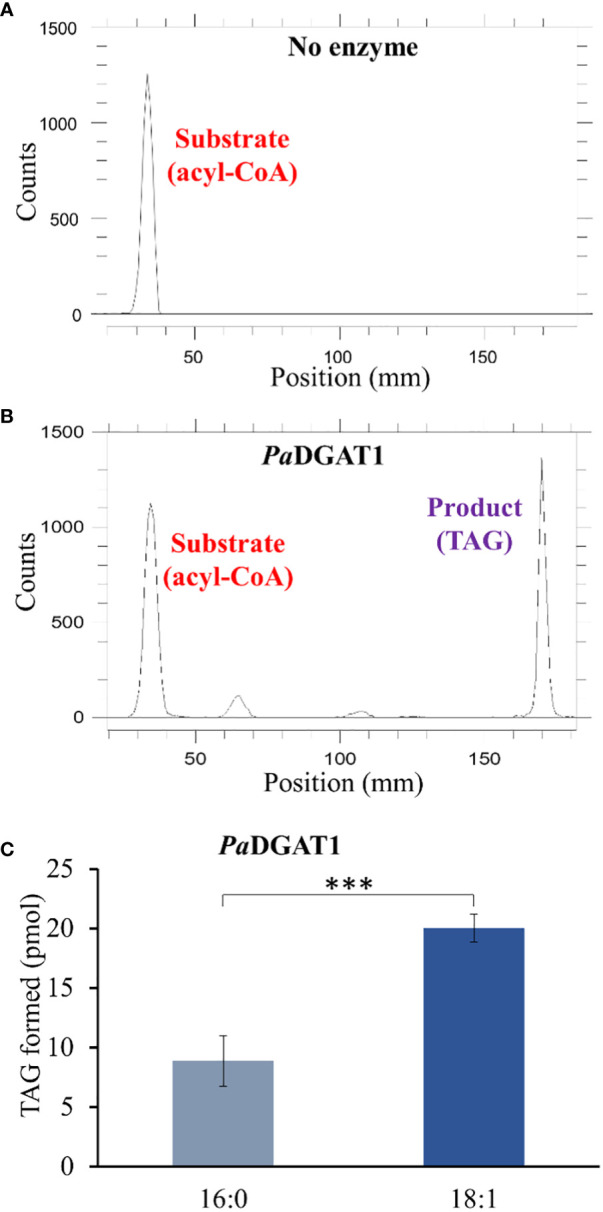
*In vitro* enzyme activity and specificity assays for yeast expressing PaDGAT1. PaDGAT1 with an N-terminal HA epitope tag was expressed in the H1246 yeast strain and was induced for 24 h. Microsomal fractions were prepared and incubated with radiolabeled fatty acyl-CoA and DAG. Saturated (palmitoyl-CoA, 16:0-CoA) and monounsaturated (oleoyl-CoA, 18:1-CoA) acyl donor substrates were tested. Representative chromatogram generated by Radio-TLC imaging scanner for negative control with only acyl-CoA substrate and no enzyme **(A)**, and PaDGAT1 microsomes incubated with 16:0 or 18:0 acyl-CoA and diolein that resulted in TAG synthesis **(B)**. Quantification of TAG product peaks revealed that PaDGAT1 prefers 18:1-CoA over 16:0-CoA **(C)**. ***P<0.001.

**Figure 8:**
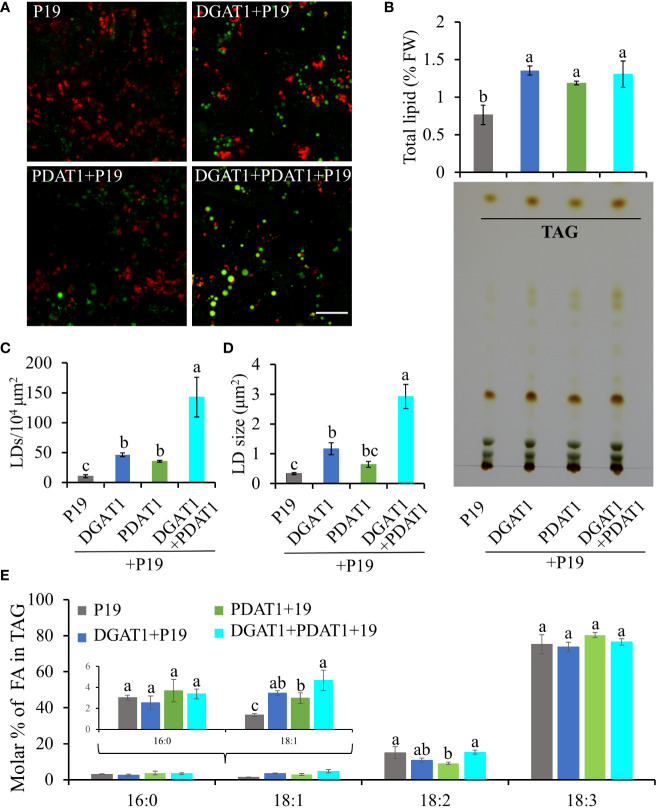
Quantification of LD accumulation and fatty acid profile in TAG in N. benthamiana leaves co-expressing PaDGAT1 and PaPDAT1. Confocal images of the accumulated LDs stained with Nile Red (green) in N. benthamiana leaves expressing control (+P19), DGAT1 (+P19), PDAT1 (+P19), and DGAT1 + PDAT1 (+P19) **(A)**. The scale bar represents 20 μm. Quantification of total lipid extracted from the leaves and separation of TAG by TLC **(B)**. Number of accumulated LDs per unit area **(C)** and their average size (area) **(D)**. Fatty acid profile of extracted TAG from the TLC plates **(E)**. Data represent mean ± SD of three independent experiments and different letters indicate significant differences (P < 0.05), as determined by ANOVA with Tukey’s post-test.

The correct figures for **Figures 5**, **8** can be seen below.

The publisher apologizes for these mistakes. The original article has been updated.

