# Supplementary figures and images for: Acyl-CoA-dependent and acyl-CoA-independent avocado acyltransferases positively influence oleic acid content in nonseed triacylglycerols
Source: Front Plant Sci. 2023 Jan 11;13:1056582. doi: 10.3389/fpls.2022.1056582 (PMC9874167; doi:10.3389/fpls.2022.1056582)

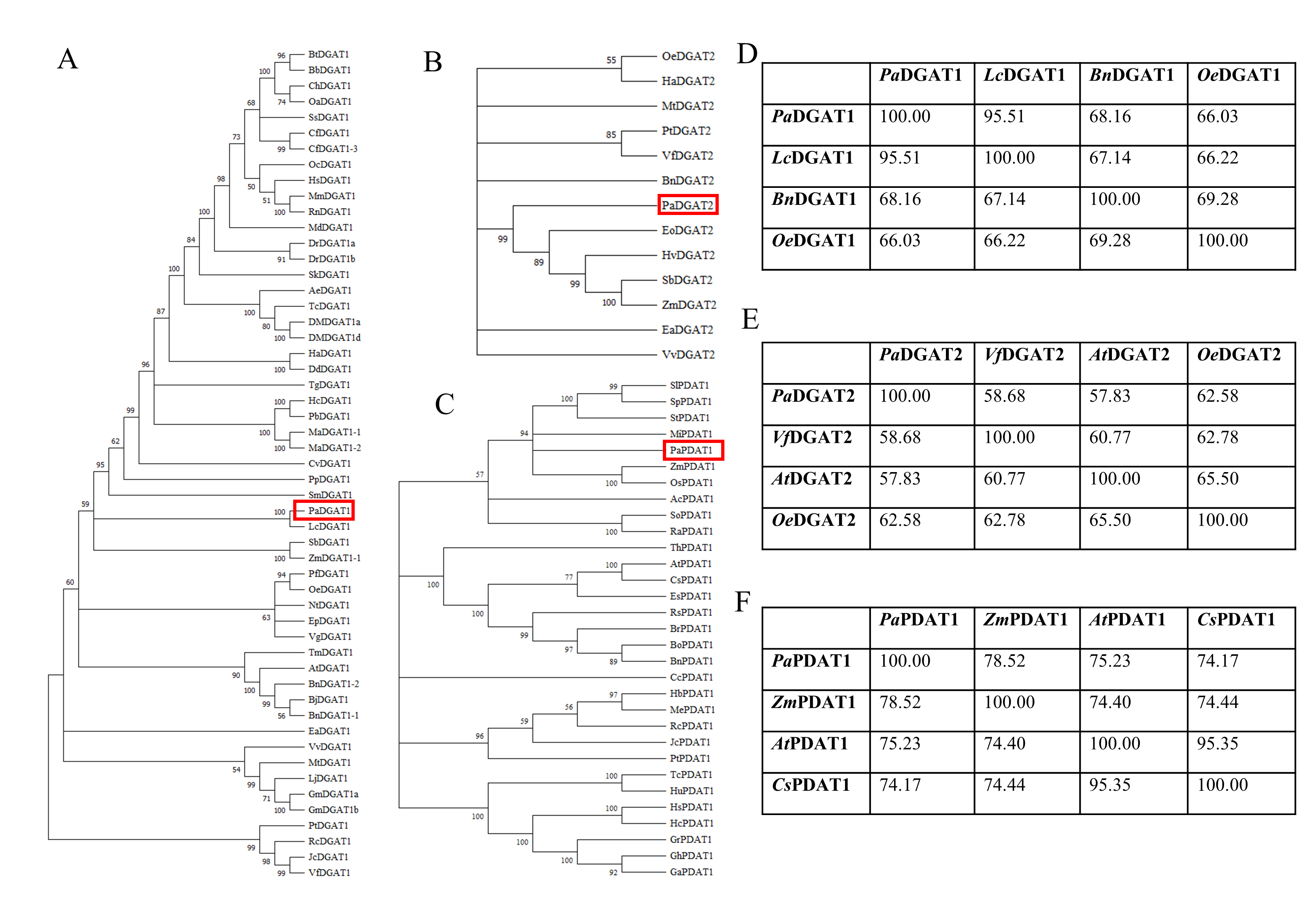

Supplement: Supplementary Figure 1 — Phylogenetic analysis and percentage sequence similarities of avocado DGAT1, DGAT2, and PDAT1 to orthologous plant proteins. Phylogenetic analysis using MEGA X software. [file Image_1.tif]

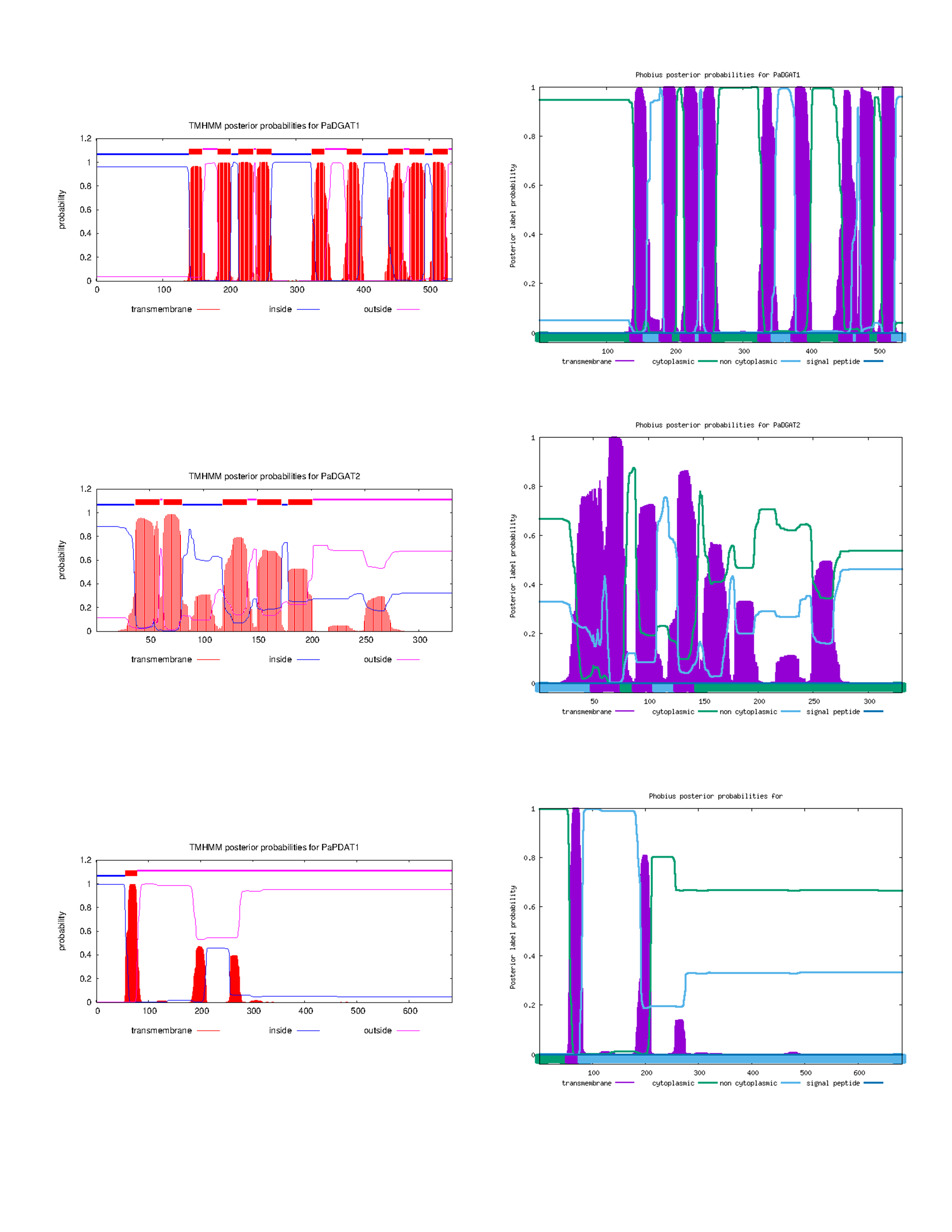

Supplement: Supplementary Figure 2 — Predicted avocado DGAT1, DGAT2, and PDAT2 membrane topologies by TMHMM and Phobius. [file Image_2.tif]

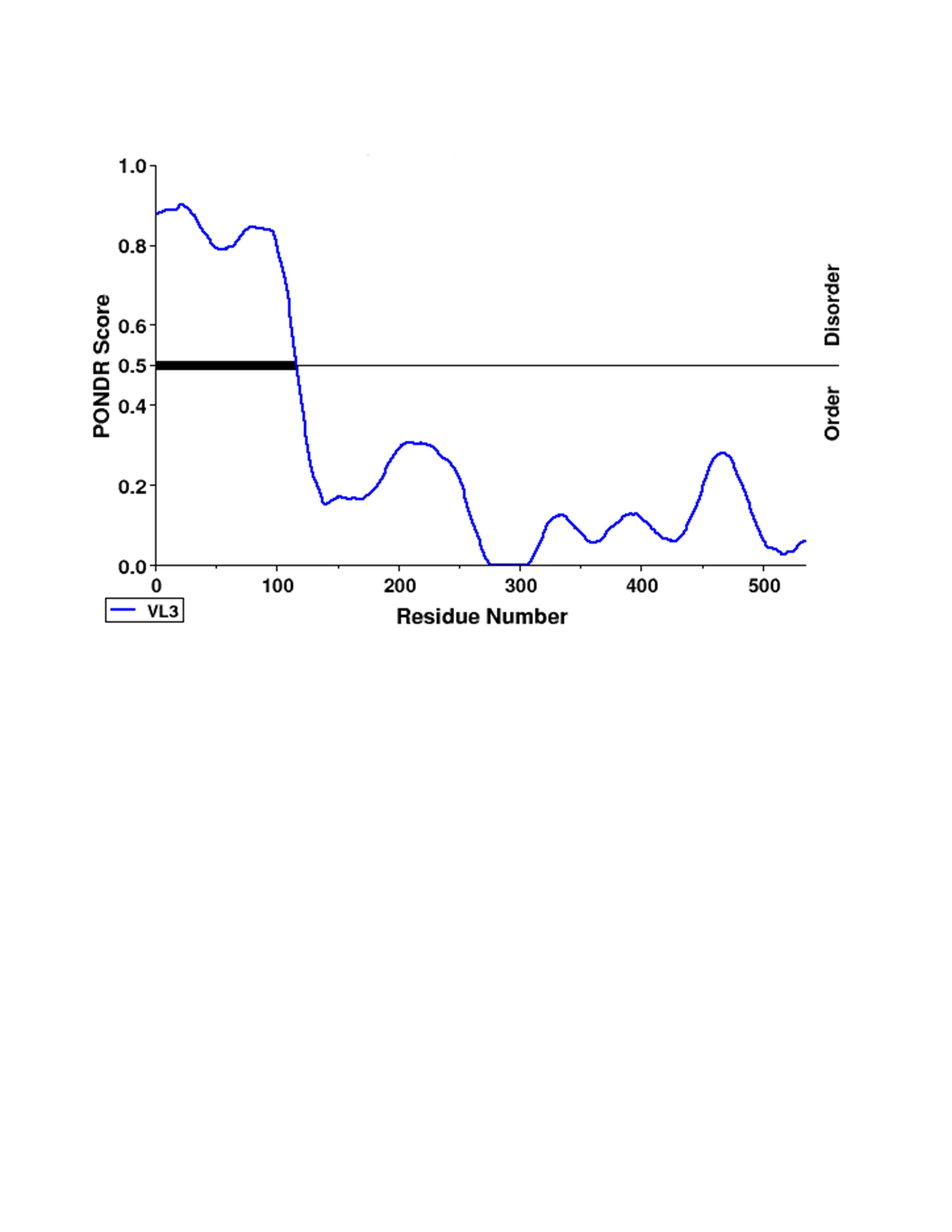

Supplement: Supplementary Figure 3 — Predicted intrinsically disordered region (IDR) in the N-terminal region of PaDGAT1. [file Image_3.tif]

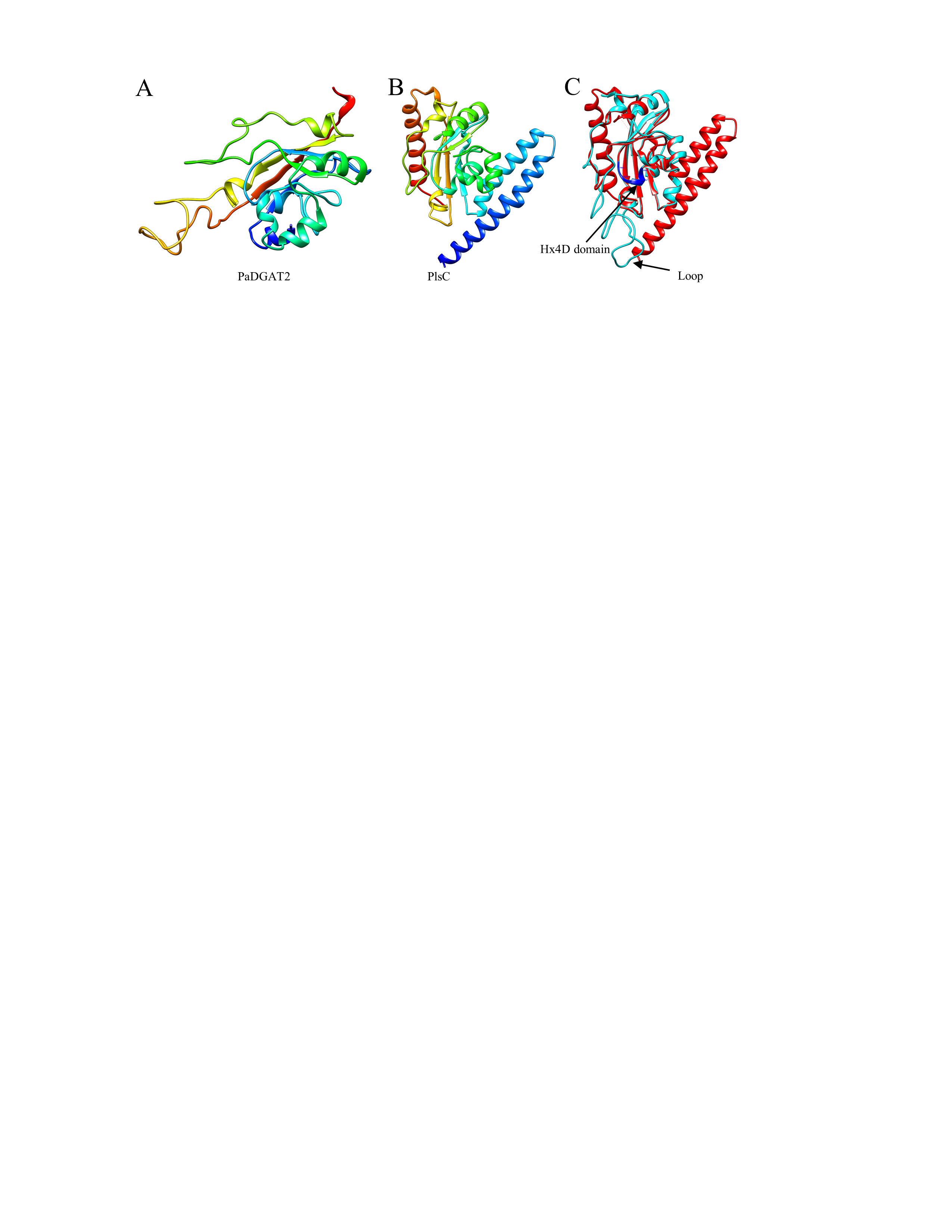

Supplement: Supplementary Figure 4 — Predicted three-dimensional (3D) structures of PaDGAT2 by the SWISS-MODEL interactive workspace tools, using the solved or predicted models for Thermotoga maritima 1-acyl-sn-glycerol-3-phosphate acyltransferase (PlsC) (PDB Id: 5kym). The 3D structures of PaDGAT2 (A) and PlsC (B) followed by their structural comparison (C) showing the PaDGAT2 in cyan, PlsC in red and Hx4D domain in blue, respectively. [file Image_4.tif]

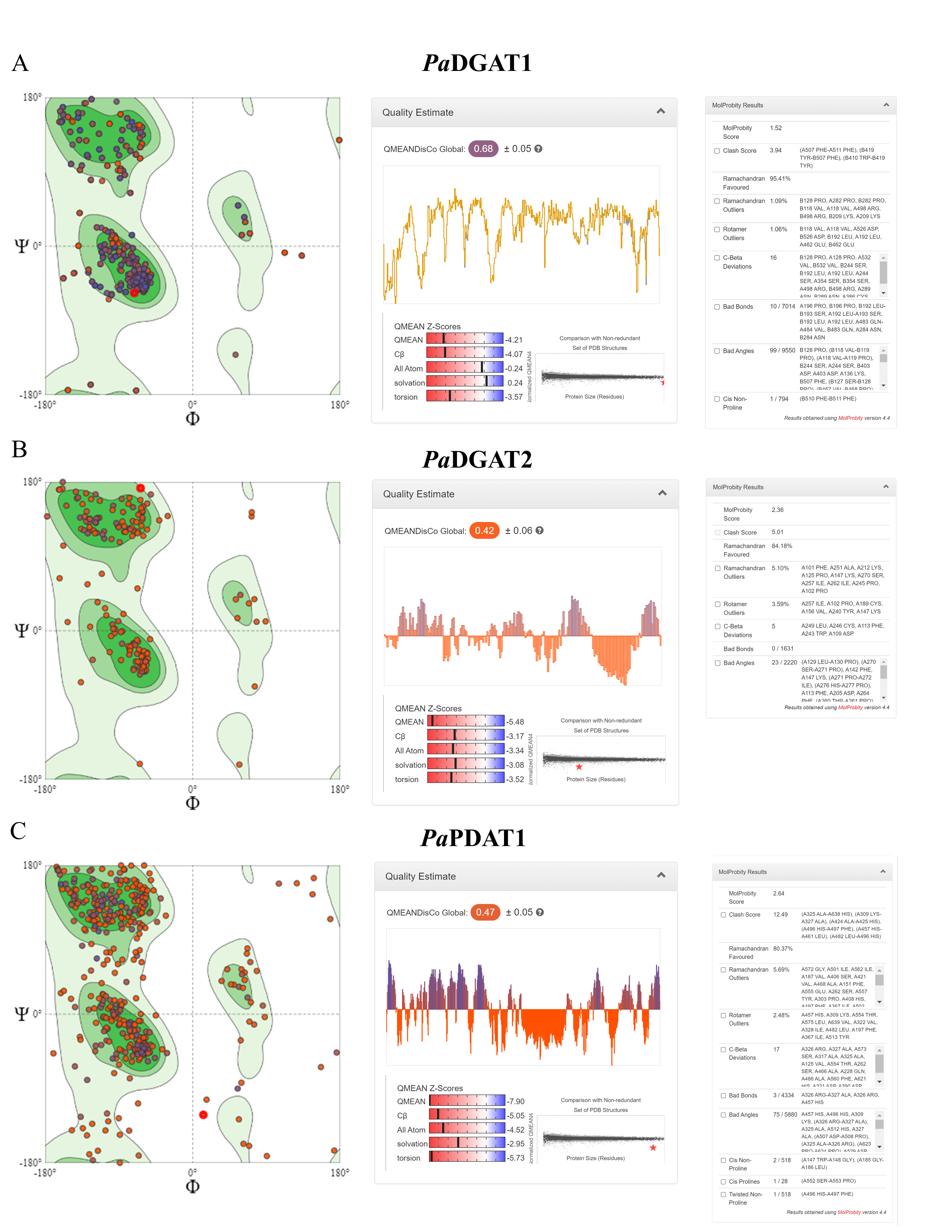

Supplement: Supplementary Figure 5 — The quality of the predicted 3D structures based on the structure assessment. The Ramachandran plot showing the bond angles (Φ and Ψ) for amino acids at each position, the quality of the predicted structure assessed by QMEANDisCo and MolProbity for PaDGAT1 (A), PaDGAT2 (B), and PaPDAT1 (C) are shown. [file Image_5.tif]

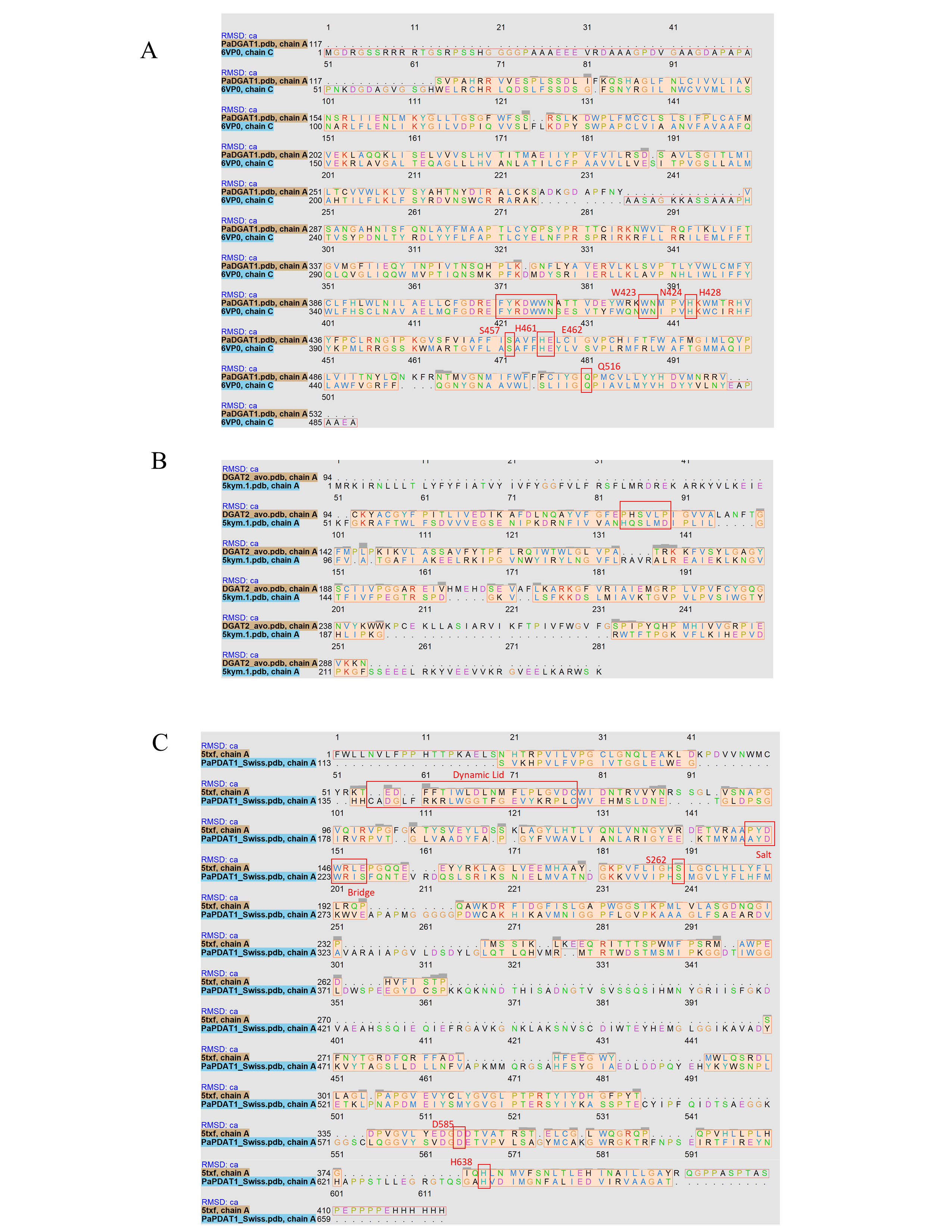

Supplement: Supplementary Figure 6 — RMSD values of structure-based sequence alignments of PaDGAT1, PaDGAT2, and PaPDAT1 with their respective templates. [file Image_6.tif]

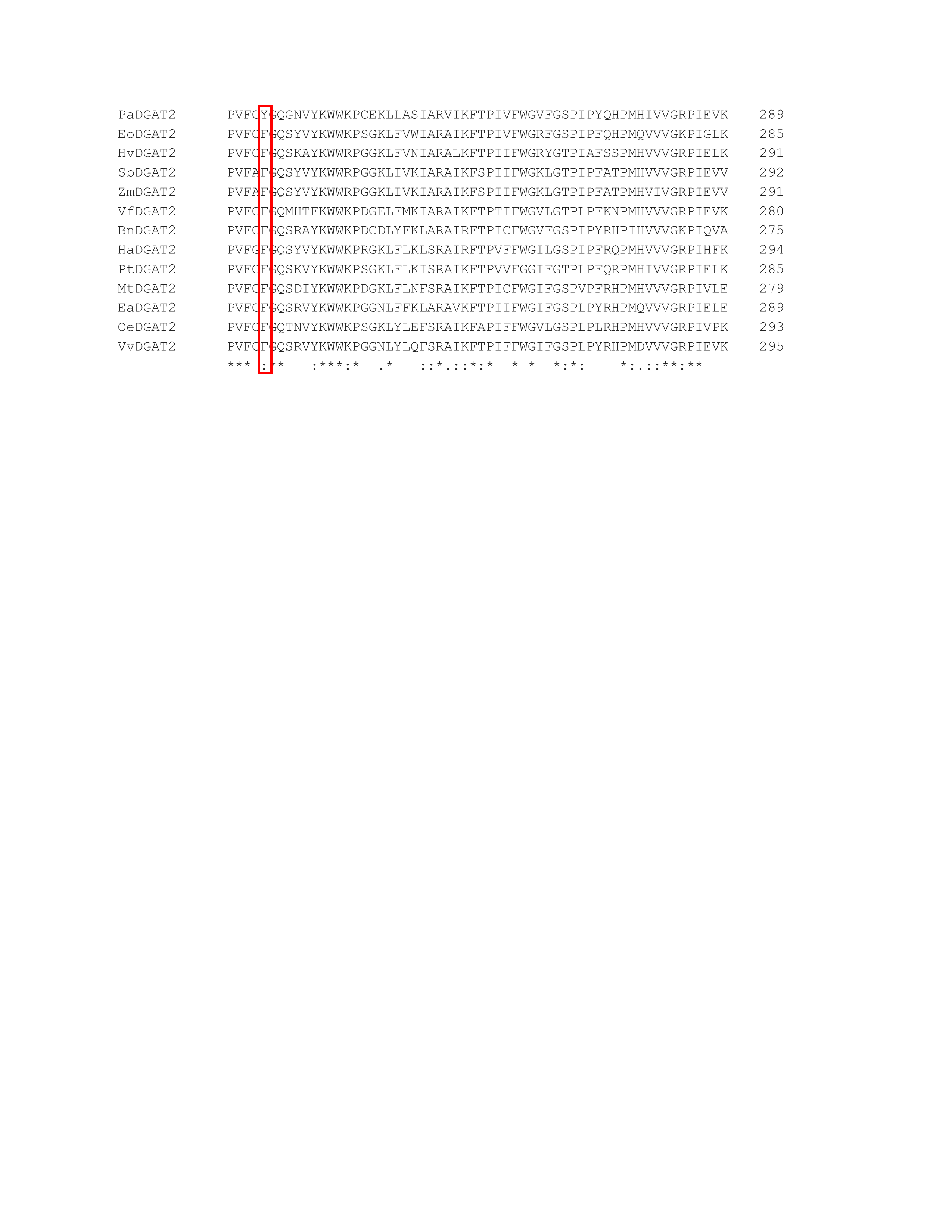

Supplement: Supplementary Figure 7 — Sequence alignment of C-terminal regions of additional plant DGAT2 proteins. The invariant Phe residue that has been changed to a Tyr residue in avocado DGAT2 (shown in red) is boxed. [file Image_7.tif]
