# Supplementary material for: Acyl-CoA-dependent and acyl-CoA-independent avocado acyltransferases positively influence oleic acid content in nonseed triacylglycerols
Source: Front Plant Sci. 2023 Jan 11;13:1056582. doi: 10.3389/fpls.2022.1056582 (PMC9874167; doi:10.3389/fpls.2022.1056582)
Supplement: Supplementary file 8 [file Table_1.docx]

**Supplementary Table 1**. Analysis of fatty acid profile of total lipids from *N. benthamiana* leaves expressing *PaDGAT1* and *PaPDAT1.* Fatty acids were extracted from *Agro*-infiltrated leaf discs, then analyzed and quantified by GC-FID as described in methods. Values represent molar percentage composition for each fatty acid in the total lipid and expressed as mean±SD of three independent experiments.

| **Fatty acid** | **WT** | **P19** | ***Pa*DGAT1** | ***Pa*DGAT1**  **+P19** | ***Pa*PDAT1** | ***Pa*PDAT1**  **+P19** |
| --- | --- | --- | --- | --- | --- | --- |
| **C16:0** | 18.24±1.10 | 15.61±0.59 | 17.80±1.27 | 16.77±1.01 | 18.39±1.81 | 17.04±2.17 |
| **C16:1** | 3.74±0.32 | 3.05±0.005 | 2.74±0.43 | 2.69±0.39 | 2.37±0.37 | 2.11±1.58 |
| **C18:1** | 0.72±1.10 | 1.40±0.15 | 3.24±0.89 | 3.71±0.77 | 3.68±0.39 | 3.16±0.74 |
| **C18:2** | 3.38±5.23 | 7.82±0.68 | 9.45±0.29 | 7.29±0.49 | 8.15±0.99 | 7.82±1.13 |
| **C18:3** | 68.78±1.14 | 69.32±0.27 | 65.47±0.65 | 68.44±1.07 | 67.14±1.65 | 67.91±1.63 |
