# Supplementary material for: Acyl-CoA-dependent and acyl-CoA-independent avocado acyltransferases positively influence oleic acid content in nonseed triacylglycerols
Source: Front Plant Sci. 2023 Jan 11;13:1056582. doi: 10.3389/fpls.2022.1056582 (PMC9874167; doi:10.3389/fpls.2022.1056582)
Supplement: Supplementary file 9 [file Table_2.docx]

**Supplementary Table 2**. Fatty acid profile of TAG from *N. benthamiana* leaves co-expressing *PaDGAT1* and *PaPDAT1.* Total lipids were extracted from *Agro*-infiltrated leaf discs, then TAG was separated by TLC followed by quantification by GC-FID as described in methods. Values represent molar percentage composition for each fatty acid in the TAG and expressed as mean±SD of three independent experiments.

| **Fatty Acid** | **P19** | **DGAT1+P19** | **PDAT1+P19** | **DGAT1+PDAT1+P19** |
| --- | --- | --- | --- | --- |
| **C16:0** | 3.02± 0.23 | 2.53±0.63 | 3.67±1.05 | 3.37±0.47 |
| **C18:1** | 1.36±0.13 | 3.44±0.23 | 2.97±0.50 | 4.66±0.97 |
| **C18:2** | 15.03±3.30 | 10.87±1.20 | 9.11±0.63 | 15.30±1.09 |
| **C18:3** | 75.21±5.29 | 73.73±2.67 | 80.33±1.39 | 76.53±1.72 |
